# Spatiotemporal mapping of CD112 unveils its dichotomous role in T-cell exhaustion and ferroptosis crosstalk in cervical cancer

**DOI:** 10.1038/s41419-026-08902-y

**Published:** 2026-05-27

**Authors:** Lihua Chen, Wenyu Lin, Yuxuan Huang, Chen Li, Ming Du, Yang Liu, Weidi Wang, Duancheng Tian, Pengming Sun, Yang Xiang

**Affiliations:** 1 Department of Obstetrics and Gynecology, Peking Union Medical College Hospital, Chinese Academy of Medical Sciences, Peking Union Medical College, Beijing, The People’s Republic of China; 2 National Clinical Research Center for Women’ s Health and Obstetric and Gynecologic Diseases,Peking Union Medical College Hospital, Chinese Academy of Medical Sciences, Peking Union Medical College, Beijing, The People’s Republic of China; 3 Laboratory of Gynecologic Oncology, Department of Gynecology, Fujian Maternity and Child Health Hospital, College of Clinical Medicine for Obstetrics & Gynecology and Pediatrics, Fujian Medical University, Fuzhou, Fujian The People’s Republic of China; 4 Fujian Key Laboratory of Women and Children’s Critical Diseases Research, Fuzhou, Fujian The People’s Republic of China

**Keywords:** Cancer microenvironment, Prognostic markers

## Abstract

CD112 emerges as a novel immune checkpoint (IC) in various malignancies, its spatial biology and dual mechanisms in cervical cancer (CC) progression are unexplored. This study systematically analyzes the dual-functions of CD112 in immune evasion and metabolic adaptation in CC. Proteomics and immune checkpoint analysis were performed on 10 CC and normal tissues to explore key targets. Multiplex immunofluorescence (mIHC) on 318 CC tissue microarrays analyzed CD112/CD112R spatial distribution and prognosis. Tumor cell and T cell co-culture models and immunocompetent murine models were used to validate effects of anti-CD112 mAb±anti-PD-1 treatment. CD112 expression in the infiltrated and margine areas correlated with PD-1^+^CD8^+^ T cells density (*r* = 0.545, *P* < 0.001), predicting unfavorable prognosis. Functional studies show that CD112 on tumor cells interacts with CD112R on CD8^+^ T cells, promoting abnormal numbers and functions of CD8^+^T cells, while CD112 antibodies can reverse CD8^+^ T cell exhaustion and enhance the anti-tumor effects by synergized with PD-1 inhibitors. Mechanistically, the transcription factor Sp1 drives the overexpression of CD112 in CC cells. Additionally, CD112 confers resistance to ferroptosis in CC through the SLC7A11/GPX4 pathway. CD112 acts as a “dual-target” mediating immune suppression and ferroptosis resistance. Combining CD112 and PD-1 inhibition may enhance CC immunotherapy.

## Introduction

Cervical cancer (CC) is the fourth most common cancer among women worldwide. In 2022, there were 662,000 newly cases worldwide, resulting in 349,000 deaths. In China, about 151,000 new cases were reported, leading to 56,000 deaths [1]. The treatment plan for early-stage cervical cancer primarily involves surgical intervention, which generally has a good prognosis. Despite significant advancements in the prevention, screening, diagnosis, and treatment of CC, recurrent or metastatic CC remains a major cause of death among cervical cancer patients. Importantly, 37% of patients diagnosed with locally advanced CC (LACC) and 14% with high-risk LACC are in China [2]. Therefore, there is a high incidence and disease burden in the country. Concurrent chemoradiotherapy (CCRT) is the preferred treatment for LACC, with a five-year survival rate of over 60% [3]. However, approximately 30% of LACC patients experience recurrence or metastasis after receiving CCRT treatment, resulting in very low survival rates [4, 5]. The heterogeneity of patients with recurrent and metastatic cervical cancer is significant, and there is currently no standard treatment protocol, highlighting the urgent need for new treatment methods and approaches.

The application of tumor immunotherapy is a significant milestone in cancer treatment, achieving great success in preclinical and clinical research [6, 7]. Immune checkpoint inhibitors (ICIs), represented by targeted therapies against Programmed Cell Death Receptor 1 (PD-1), Programmed Cell Death Ligand 1 (PD-L1), or Cytotoxic T Lymphocyte Antigen 4 (CTLA-4), are currently hot topics and focal points in the field of cancer research. However, due to primary or acquired resistance, only a small portion of patients benefit from ICI treatment [8]. Currently, the main research focus of cervical cancer immunotherapy is on advanced, recurrent, or metastatic cervical cancer. The Keynote-158 study [9] indicated that the objective response rate (ORR) of PD-1 inhibitor (pembrolizumab) treatment for cervical cancer is only 14.3%. The KEYNOTE-826 study [10] showed that pembrolizumab combined with platinum-based chemotherapy significantly extended progression-free survival (PFS) and overall survival (OS) in first-line treatment for cervical cancer. Based on these two studies, the 2022 NCCN cervical cancer guidelines included the immunotherapy plus chemotherapy ± bevacizumab regimen in the first-line treatment recommendations for advanced cervical cancer (in the PD-L1 positive population) [11]. Although immunotherapy for cervical cancer has progressed from later lines to second-line and even first-line treatment, the efficacy of PD-1 and PD-L1 inhibitors as monotherapy is not very satisfactory. Immunotherapy faces the issue of low response rates in CC treatment, resulting in most patients not benefiting from it. Furthermore, both second-line and first-line treatments are limited to PD-L1-positive patients. Therefore, it is an urgent need to explore effective new immune checkpoints and their molecular mechanisms, accurately identify potential beneficiary populations, and improve the efficacy of immunotherapy.

CD112 and its receptor (CD112R) are both new immune checkpoint targets. CD112 (Nectin-2), also known as Poliovirus Receptor Related 2 (PVRL2), belongs to the Nectin protein family. Studies have shown that CD112 plays an important role in the occurrence and development of tumors. Relevant research indicates that its expression is elevated in various malignant tumors, such as lung cancer, ovarian cancer, and breast cancer [12–14]. Furthermore, Kučan Brlić P et al. [15] suggested that CD112 is also involved in the regulation of immune responses. Further studies have shown that CD112 is similar to the poliovirus receptor (PVR) and can bind to CD226 or TIGIT expressed on CD8^+^ T cells and NK cells to generate anti-tumor responses, thereby promoting tumor development by inhibiting immune cell-mediated cytotoxic responses [16]. CD112R, also known as PVR Related Immunoglobulin Domain Containing (PVRIG), is a co-inhibitory receptor belonging to the PVR family, first reported as a new inhibitory receptor in 2016 [17]. CD112R is highly expressed in malignant tumors such as renal cancer, ovarian cancer, lung cancer, prostate cancer, and endometrial cancer, and is primarily expressed on the surface of NK cells and T cells in various types of solid tumors [18, 19]. In humans, CD112 is the only ligand that can bind to CD112R [17]. Turnis et al. [19] found that blocking CD112R promotes the production of interferon-γ (IFN-γ) in tumor-infiltrating immune cells, with efficacy similar to blocking TIGIT or PD-1. Murter et al. [20] found that CD112R knockout mice exhibited suppressed tumor growth compared to wild-type mice, with an increase in tumor-infiltrating immune cells, particularly CD8 + T cells, in the tumor tissues of the knockout mice. Importantly, CD112 is expressed in both PD-L1-negative and PD-L1-positive tumors in breast, ovarian, and lung tissues [19]. Therefore, the CD112/CD112R axis can inhibit T cell immune function in PD-L1 negative tumors, and targeting the blockade of the CD112/CD112R axis may be used to treat PD-L1 negative tumors or tumors that are resistant to other immune checkpoint inhibitors. Thus, the CD112/CD112R axis is a potential target for immunotherapy and is an ideal target for combination therapy with PD-1 inhibitors.

To determine the role and potential molecular mechanisms of the novel immune checkpoint CD112/CD112R axis in the progression of cervical cancer and tumor immune evasion, we validated the high expression of the novel immune checkpoint CD112/CD112R axis in cervical cancer through proteomics, tissue microarrays, and in vivo/vitro experiments, and found it to be associated with poor prognosis. The transcription factor SP1 promotes the transcription of CD112. CD112 binds to CD112R on the surface of CD8^+^ T lymphocytes, inhibiting the proliferation and secretion function of CD8^+^ T lymphocytes, thereby mediating an immune-evasive tumor microenvironment. And blocking the CD112/CD112R axis can restore CD8^+^ T lymphocyte function and produce significant anti-tumor effects. Also, CD112 may activate the SLC7A11/GPX4 pathway, which reduces the sensitivity of cervical cancer cells to ferroptosis. Therefore, CD112/CD112R axis could be a new immuno-therapeutic targets for patients with advanced, recurrent, or metastatic cervical cancer.

## Results

### High expression of the novel immune checkpoint CD112 in cervical cancer is associated with CD8 + T cell exhaustion

**Fig. 1:**
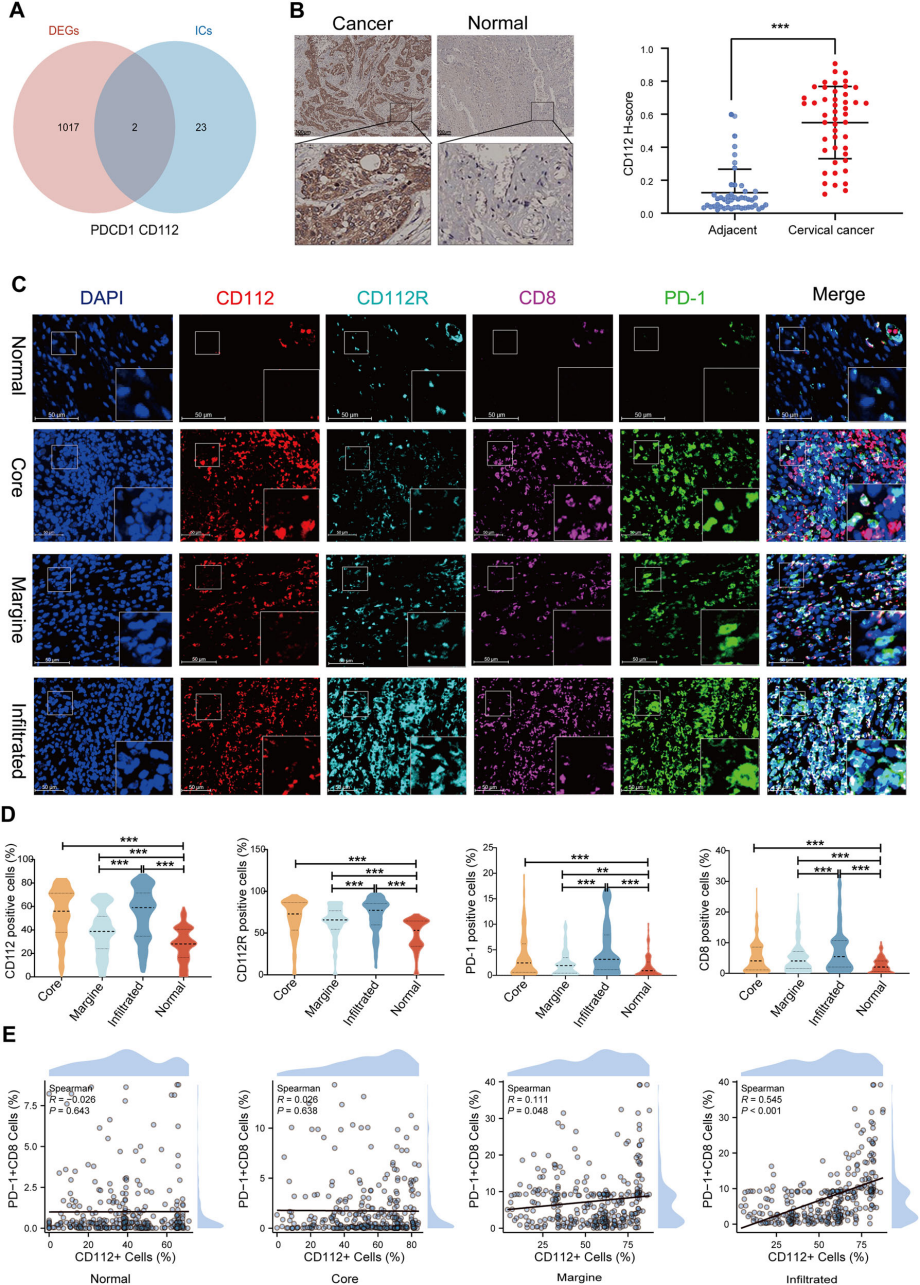
Novel immune checkpoint CD112 was highly expressed in cervical cancer and associated with CD8^+^T cell exhaustion. **A** Combined analysis of differential protein and novel immune checkpoints in cervical cancer; **B** IHC staining of CD112 in 46 CC samples from PUMCH. Correlations were analyzed by Chi-square test (scale bars = 100 μm); **C**, **D** Multiplex immunohistochemical was used to detect the expression of CD112, CD112R, PD-1 and CD8 in different tumor regions of cervical cancer tissue microarray (scale bars = 50 μm); **E** Spearman correlation coefficient was used to detect the correlation between CD112^+^ cells and PD-1^+^ CD8^+^ T cells in different tumor regions of sample tissues. All experiments were performed three times, and data are presented as mean ± SD. ns non significant; *p < 0.05; **p < 0.01; ***p < 0.001.

Immunotherapy monotherapy faces low response rates in CC treatment, which means most patients don’t benefit from it [9, 10]. Therefore, we explored potential novel immune checkpoints (ICs) in CC. We identified the expression profiles of aberrantly regulated proteins in 10 pairs of CC as compared with adjacent cohorts (Figure S1). We found that 689 proteins were upregulated and 330 proteins down-regulated in CC. Recently, more and more novel ICs have been discovered, which we’ve summarized in Table S1. By combining the differential proteins and the aforementioned ICs, we revealed that CD112 and PD-L1 were both highly expressed in CC (Fig. 1A). To further verify the expression level of CD112 in CC, we examined 46 pairs of CC and adjacent tissues and found that the expression of CD112 was significantly increased in cervical cancer(Fig. 1B).

CD112 and its receptor (CD112R) are both new ICs. It has been reported that blocking CD112R promotes the production of interferon-γ (IFN-γ) in tumor-infiltrating immune cells, with effects similar to blocking TIGIT or PD-1 [17]. Therefore, we examined the expression of CD112, CD112R, PD-1 and CD8 in different tumor regions, such as tumor core, tumor margine and tumor-infiltrated area and normal tissues in 318 patients with tissue microarrays. To investigate whether the expression of CD112 in cervical cancer tissues functionally inhibits CD8 + T cell immunity in TME. In all samples, the ratios of CD112 + , CD112R + , CD8 + , and PD-1+ cells in the core, margine, and infiltrated areas were higher than the normal tissues, with the highest expression levels of those indicators in the infiltrated area (Fig. 1C, D). Notably, statistical analysis showed a strong positive correlation between CD112+ cells and PD-1 + CD8 + T cells in the core, margin, and infiltrated areas, but there wasn’t in adjacent tissues. (Fig. 1E). Taken together, these results suggest that CD112 is highly expressed in CC and may contribute to tumor progression by promoting CD8 + T cell exhaustion.

### High CD112+cells, PD-1 + CD8 + T cell and CD112R + CD8 + T cell infiltration correlate with poor CC prognosis

**Fig. 2:**
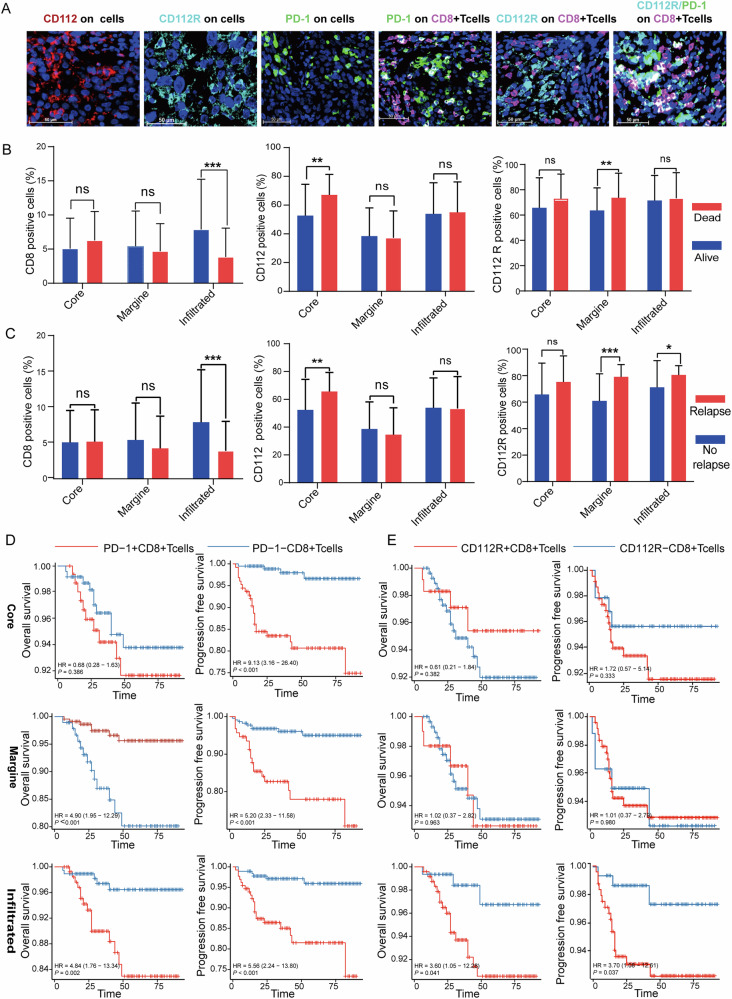
High CD112+cells, PD-1 + CD8 + T cell and CD112R + CD8 + T cell infiltration correlate with poor CC prognosis. **A** Multiplex immunohistochemical was used to detect the enrichment of CD112, CD112R, PD-1 and CD8 in cervical cancer tissue samples(scale bars = 50 μm); **B**, **C** The expression of CD8-positive, CD112-positive and CD112R-positive cells in different tumor regions of cervical cancer tissue samples, Kaplan-Meier model was used to analyze the relationship between PD-1 and CD112R expression on CD8^+^ T cells and prognosis. All experiments were performed three times, and data are presented as mean ± SD. ns non significant; *p < 0.05; **p < 0.01; ***p < 0.001.

To further validate the relationship between the expression of CD112, CD112R, PD-1, and CD8 with the prognosis of CC patients, we conducted a further analysis of the prognostic outcomes based on different expression patterns in 318 patients who underwent radical surgery for CC. Based on our observation that CD112, CD112R, PD-1, and CD8 were significantly enriched in the core and infiltrated areas (Fig. 2A), we hypothesized that the presence of CD112+ tumor cells, PD-1 + CD8 + T cells, and CD112R + CD8 + T cells would have an adverse effect on survival. Statistical analysis showed that the expression of CD8 + T cells in the infiltrated areas was significantly associated with overall survival (OS) and disease-free survival(DFS). The presence of CD112R+ cells in the infiltrated areas was significantly associated with OS, while CD112R+ cells in both the margine and infiltrated areas were significantly associated with DFS. Furthermore, CD112+ cells in the core area were significantly associated with both OS and DFS (Fig. 2B, C).

PD-1 and CD112R are primarily expressed on immune cells, so we further investigated the relationship between the expression of PD-1 and CD112R on CD8 + T cells and prognosis. The results showed that PD-1 + CD8 + T cells in the margine and infiltrated areas were associated with a lower OS. Furthermore, the presence of PD-1 + CD8 + T cells in the core, margine and infiltrated areas was associated with a lower DFS (Fig. 2D). Additionally, CD112R + CD8 + T cells in the infiltrated areas were associated with lower OS and DFS (Fig. 2E).

### Establishment of a prognostic model of CC based on immune checkpoint co-expression patterns

**Fig. 3:**
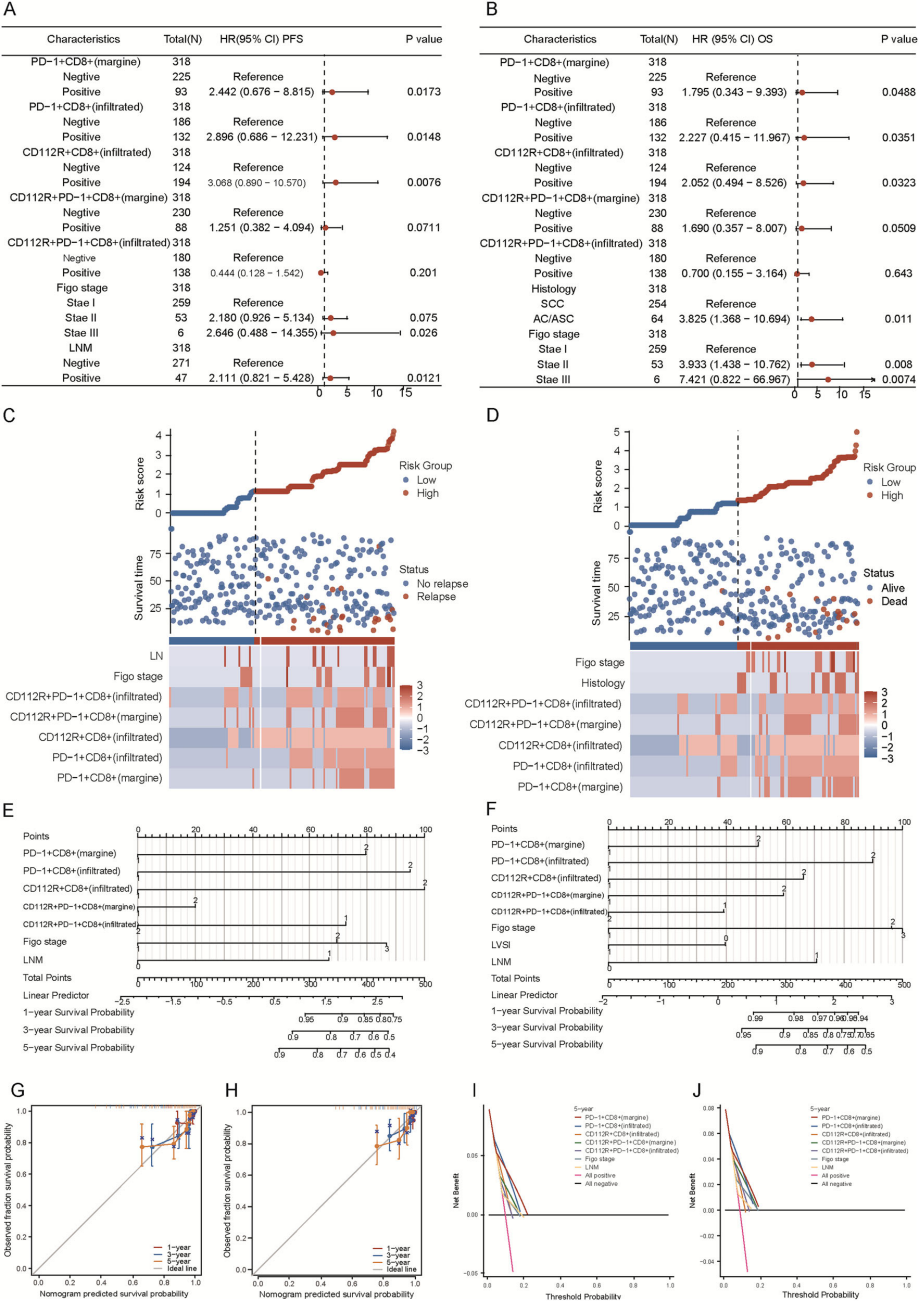
The prognostic model of CC based on immune checkpoint co-expression patterns. **A** Multivariate Cox regression analysis for progression-free survival was used to analyze the risk degree of multiple immune checkpoint combined subtypes in different tumor regions of cervical cancer; **B** Multivariate Cox regression analysis for overall survival was used to analyze the risk degree of multiple immune checkpoint combined subtypes in different tumor regions of cervical cancer. **C**, **D** Overall distribution of risk scores, survival status, and gene expression profiles of different immune checkpoint co-expression patterns in relapsed and non-relapsed populations, dead and surviving populations. **E**, **F** Nomogram models for progression-free survival (PFS) and overall survival (OS) based on various clinical and immunological factors. **G**, **H** The model’s calibration curves for progression-free survival (PFS) and overall survival (OS) based on various clinical and immunological factors. **I**, **J** The DCA curves for progression-free survival (PFS) and overall survival (OS) based on various clinical and immunological factors.

We analyzed the presence of potential combined subtypes that have not yet been identified and could serve as biomarkers for pre-treatment stratification of patients with CC. Multivariate Cox regression analysis for PFS showed that CD112R + CD8 + T cell (infiltrated) had the highest HR (HR = 3.068, 95%CI = 0.890-10.570, *p* = 0.0076), followed by PD-1 + CD8 + T (infiltrated) (HR = 2.896, 95% CI = 0.686-12.231, *p* = 0.0148), higher FIGO stage (HR = 2.646, 95% CI = 0.488-13.355, *p* = 0.026), PD-1 + CD8 + T (margine) (HR = 2.442, 95% CI = 0.676-8.815, *p* = 0.0173), LNM (HR = 2.111, 95% CI = 0.821-5.428, *p* = 0.0121) (Fig. 3A). As for OS, higher FIGO stage had the highest HR (HR = 7.421, 95% CI = 0.822-66.967, *p* = 0.0074), followed by AC/ASC (HR = 3.825, 95% CI = 1.368-10.694, p = 0.011), PD-1 + CD8 + T (infiltrated) (HR = 2.227, 95% CI = 0.415-11.967, *p* = 0.0351), CD112R + CD8 + T(infiltrated) (HR = 2.052, 95% CI = 0.494-8.526, *p* = 0.0323) and PD-1 + CD8 + T (maigine) (HR = 1.795, 95% CI = 0.343-9.393, *p* = 0.0488) (Fig. 3B). According to Fig. 3C, D, we found that the different co-expression patterns of CD112R, PD-1 and CD8 could significantly distinguish the relapsed and non-relapsed populations, and the dead and surviving populations. Finally, for PFS and OS, we used nomograms based on age, different patterns of immune checkpoint and immune cell co-expression, pathological type, clinical stage, myometrial invasion, parametrial invasion, LVSI, and LNM status, respectively. The VIF values of these factors ranged from 1.52–3.42, indicating that the nomogram model constructed by these factors was stable and reliable (Fig. 3E, F). Furthermore, when predicting patients ‘PFS and OS, we observed that the model’s calibration curves closely approximated the diagonal within most predicted probability intervals, indicating high accuracy in predicting PFS and OS (Fig. 3G, H). Finally, we constructed decision curve analyses for PFS and OS, which revealed that to predict clinical net benefit in progression-free survival, the DCA curves consistently remained above the reference lines representing “full treatment” and “full no treatment” across the entire threshold probability range (approximately from 0 to 0.7). Consistent with the PFS analysis, the OS DCA curves also demonstrated significantly and stably higher values than the “full treatment” and “full no treatment” reference lines over a broad threshold probability range (from near 0 to above 0.6). The DCA prognostic model developed in this study not only exhibits statistical predictive power (discrimination and calibration) but also holds clear clinical practical value (Fig. 3I, J).

### In cervical cancer, CD112 binds to CD112R and promote CD8 + T cell exhaustion

**Fig. 4:**
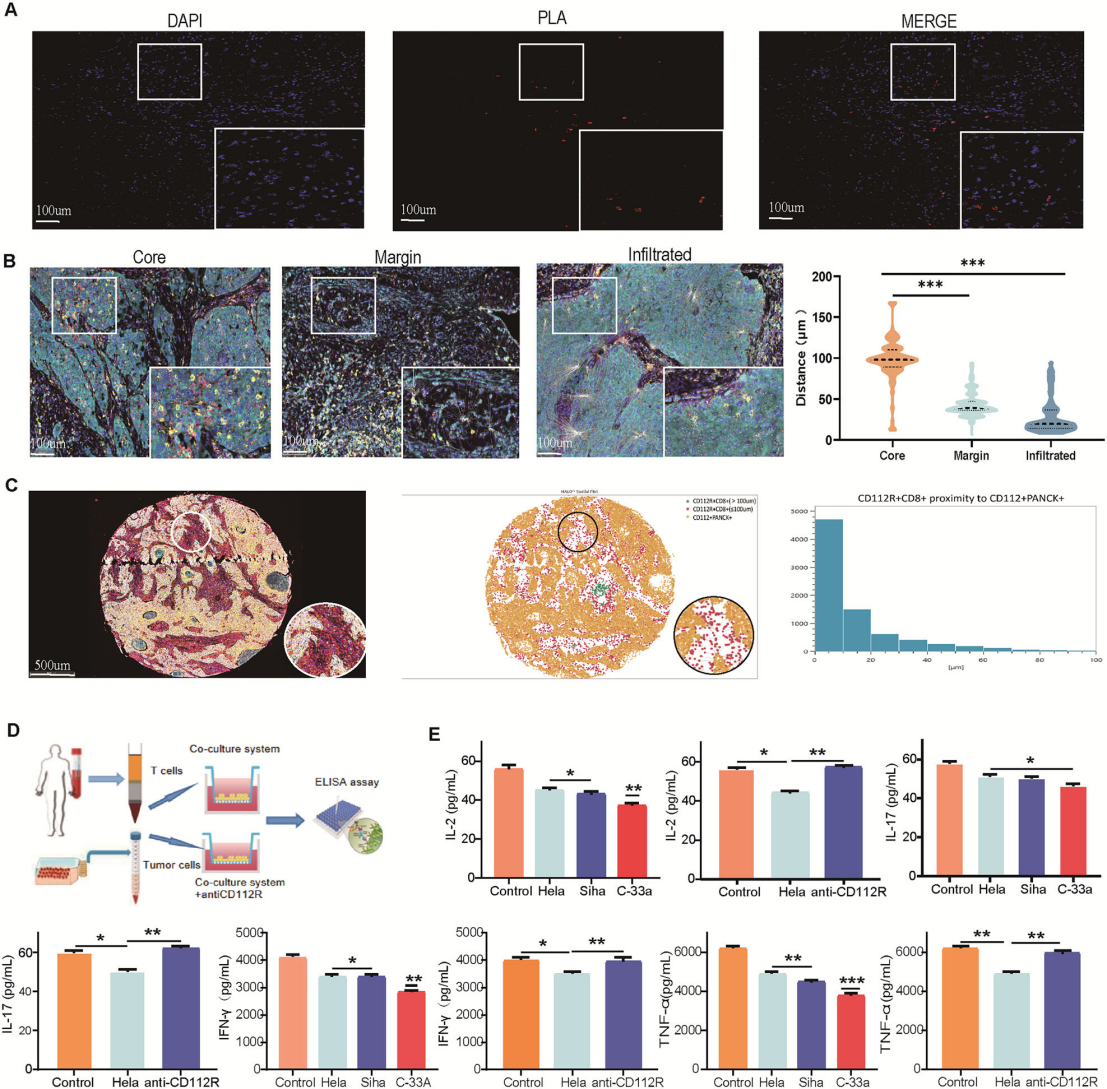
CD112 binds to CD112R and promote CD8 + T cell exhaustion in cervical cancer. **A** Representative images of PLA in the tissue of cervical cancer (scale bars = 100 μm). **B** Representative images of the distance of CD112 and CD112R in the different region of cervical cancer (such as core, infiltrated and margin)(scale bars = 100 μm) and the average spatial distance between CD112^+^ tumor cells and CD112R^+^CD8^+^T cells by corresponding quantitative bar chart. **C** Representative images of proximity analysis between CD112^+^CK^+^ cells (CD112^+^ tumor cells, yellow) and CD112R^+^CD8^+^T cells (red) and the number of CD112R^+^CD8^+^T cells distributed from CD112+ tumor cells with different distance range. **D** Schematic diagram of the co-culture system with cervical cancer cells and T cells; **E** ELISA verified the effect of cervical cancer cells inhibiting the secretion function of T cells, and the addition of CD112 inhibitors can restore the secretion function of T cells; The data are presented as mean ± SD. ns non significant; *p < 0.05; **p < 0.01; ***p < 0.001.

CD112, a member of the immunoglobulin superfamily, can bind to CD112R, which is highly expressed on tumor-infiltrating lymphocytes to produce immunosuppressive signals [16]. Related studies have shown that CD112R knockout can inhibit tumor growth and increase tumor-infiltrating immune cells, especially CD8^+^ T cells [17]. To investigate evidence of CD112-CD112R interaction in cervical cancer tissues at the tissue-in-situ level, we first detected red signals in cervical cancer tissues using Duolink™PLA detection, indicating spatial interaction between the two proteins CD112 and CD112R at the tissue-in-situ level (Fig. 4A). Our analysis of cervical cancer tissues revealed that the average spatial distance between CD112^+^ tumor cells and CD112R^+^CD8^+^T cells was significantly smaller in tumor infiltrate areas and marginal zones (32 μm and 48 μm) compared to the tumor core region (103 μm). Relevant studies suggest that spatial distances <100 μm indicate mutual interaction between the two cells[21]. Proximity analysis further demonstrated frequent co-localization and close proximity between CD112^+^CK^+^ cells (CD112^+^ tumor cells, yellow) and CD112R^+^CD8^+^ T cells (red) within the field of view. The majority of CD112R^+^CD8^+^ T cells were distributed within ≤100 μm of CD112^+^ tumor cells, with a significant counting peak observed in the 0–20 μm distance range, followed by a gradual decrease in frequency with increasing distance (Fig. 4B). In this study, we investigated whether CD112 and CD112R also mediate a suppressive TIME in CC. T lymphocytes isolated from healthy human peripheral blood were activated and co-cultured with CC cell lines (SiHa, HeLa and C-33A cells). Interleukin-2 (IL-2), interleukin-17 (IL-17), interferon-γ (IFN-γ), and tumor necrosis factor-α (TNF-α) levels were measured by ELISA (Fig. 4C). The results showed that CC cells with high expression of CD112 inhibited the ability of T cells to secrete cytokines IL-2, IL-17, IFN-γ and TNF-α. However, after adding CD112 inhibitor to the co-culture system of HeLa cells and T cells, the function of T cells secreting cytokines was restored (Fig. 4D).

### Blocking the CD112/CD112R axis could effectively inhibit tumor growth in vivo

**Fig. 5:**
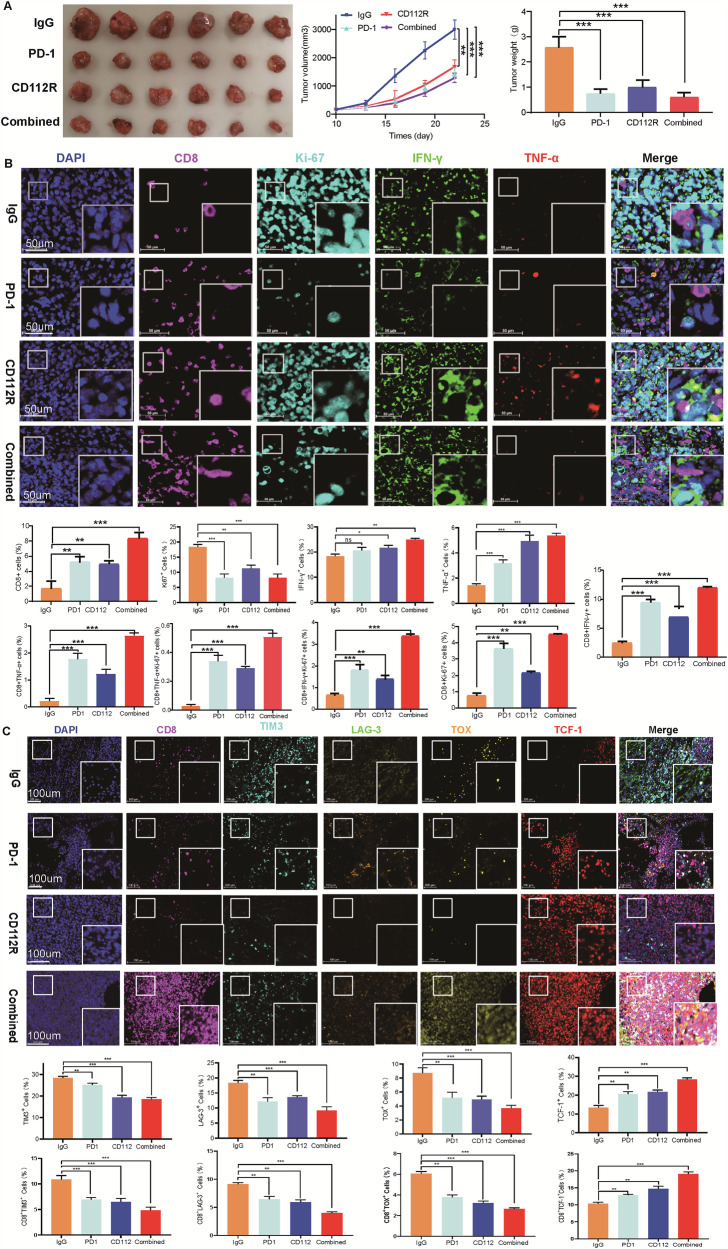
Blocking the CD112/CD112R axis remodels the tumor immune micro-environment and inhibits tumor growth in vivo. **A** Representative images of tumors taken at the end of the study and the tumor sizes in Kunming mice of the four treatment groups; **B** Representative images of multiplex immunohistochemical with panel 1 and the number and function of CD8 + T lymphocytes in tumor tissues of the four treatment groups(scale bars = 50 μm); **C** Representative images of multiplex immunohistochemical with panel 2 and the number and function of CD8 + T lymphocytes in tumor tissues of the four treatment groups(scale bars = 100 μm). All experiments were performed three times, and data are presented as mean ± SD. ns non significant; *p < 0.05; **p < 0.01; ***p < 0.001.

To verify whether blocking CD112/CD112R axis could exert anti-tumor effect in cervical cancer and whether blocking CD112/CD112R axis could enhance the anti-tumor effect of PD-1 inhibitors. We established a mouse model of CC xenografts and randomly divided them into four treatment groups. The results showed that compared with the control group, CD112 inhibitor could significantly inhibit the growth of tumors, and the CD112 inhibitor and PD-1 inhibitor could enhance the anti-tumor effect of PD-1 inhibitor (Fig. 5A). In addition, four groups of mouse tumor tissues were used for multiple IHC assays. The results showed that the infiltration of CD8^+^ T lymphocytes in the tumors was significantly increased in the CD112 inhibitor, PD1 inhibitor and combined therapy groups compared with the control group. Moreover, the infiltration level of CD8^+^ T lymphocytes in the combined therapy groups was higher than that in the CD112 inhibitor group and PD1 inhibitor group. Additionally, in the combined therapy groups, the levels of proliferating CD8^+^ T lymphocytes and the secretion levels of TNF-α and IFN-γ by CD8^+^ T lymphocytes were higher than those in the PD-1 inhibitor/CD112 inhibitor group, which in turn were higher than those in the control group (Fig. 5B). To further investigate the impact of targeted CD112 therapy on the multidimensional state of T cell exhaustion/disfunction, we performed mIHC on paraffin-embedded tumor tissue sections from mice. The results demonstrated decreased expression of TIM-3^+^CD8^+^ T, LAG-3^+^CD8^+^ T, and TOX^+^CD8^+^ T in CD112 inhibitor group and PD1 inhibitor group, and the combination therapy group compared to the control group, particularly in the combination therapy group. Conversely, TCF1^+^CD8^+^ T exhibited an opposite trend, indicating that anti-CD112 therapy reshaped the tumor immune microenvironment and enhanced anti-tumor effects (Fig. 5C).

### CD112 promotes the proliferation and invasion of cervical cancer cells

The expression level of CD112 in six cervical cancer cell lines (HeLa, SiHa, C-33A, HCC-94 and Caski) was detected (Figure S2). According to different expression levels and biological backgrounds, we selected HeLa (HPV-18 positive), SiHa (HPV-16 positive) and C-33A (HPV-negative) cells to construct CD112 knockdown and overexpression stable cell models, respectively. The changes in CD112 mRNA and protein expression levels were detected by PCR and Western blot (Figure S2). Interestingly, our clinical data results demonstrated no statistically significant correlation between CD112 expression and specific HPV genotypes (HPV-16, HPV-18, or other types) (Figure S5B).

**Fig. 6:**
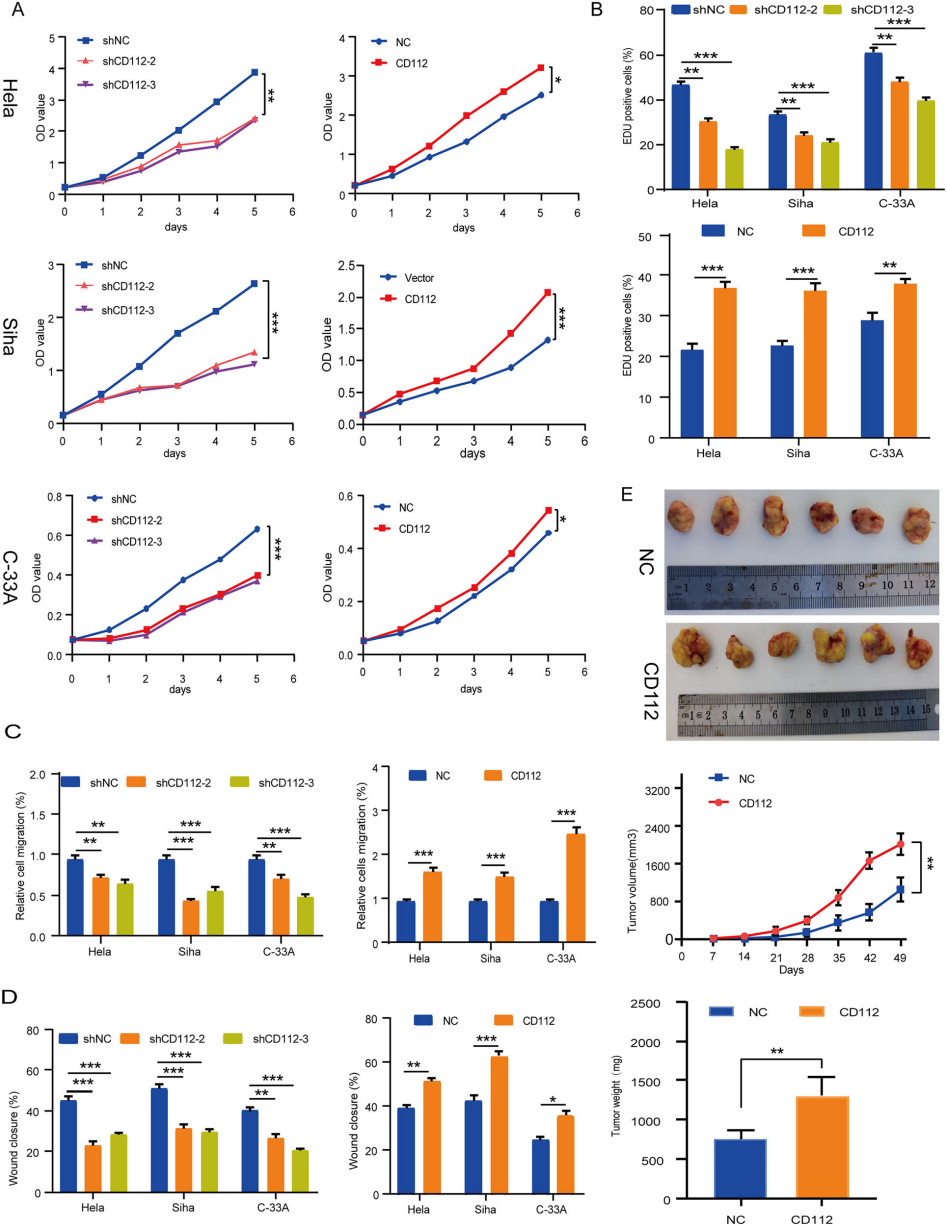
CD112 promotes CC proliferation, invasion, and migration in vitro and in vivo. **A** Growth curve assay based on CCK8 analysis in CC cells; **B** EDU proliferation assays were performed in CC cells; **C** Matrigel invasion assay was performed in CC cells; **D** Wounding healing assay was performed in CC cells; **E** Representative images and statistical analysis of subcutaneous xenograft derived from Siha cells. All experiments were performed three times, and data are presented as mean ± SD. ns non significant; *p < 0.05; **p < 0.01; ***p < 0.001.

To investigate the effect of CD112 on CC, growth curve analysis and EdU assay results showed that knockdown of CD112 significantly inhibited the proliferation of CC cells, while overexpression of CD112 significantly promoted the proliferation of CC cells (Fig. 6A, B). The wound-healing assay and transwell invasion assay showed that CD112 overexpression promoted the migration and invasion of CC cells, while CD112 knockdown inhibited their migration and invasion (Fig. 6C, D). These results suggest that increased CD112 expression levels promote CC cells' proliferation and metastasis. To evaluate the tumorigenic effect of CD112 in vivo, a mouse model of CC xenografts was established. It was found that the tumor volume and weight of mice injected with U14-CD112 cells were significantly higher than those of the control group (Fig. 6E). The above results suggest that the increased expression level of CD112 in CC cells can promote the proliferation, invasion and metastasis of tumor cells.

### Transcription factor SP1 promotes the transcription of CD112 in cervical cancer cells

**Fig. 7:**
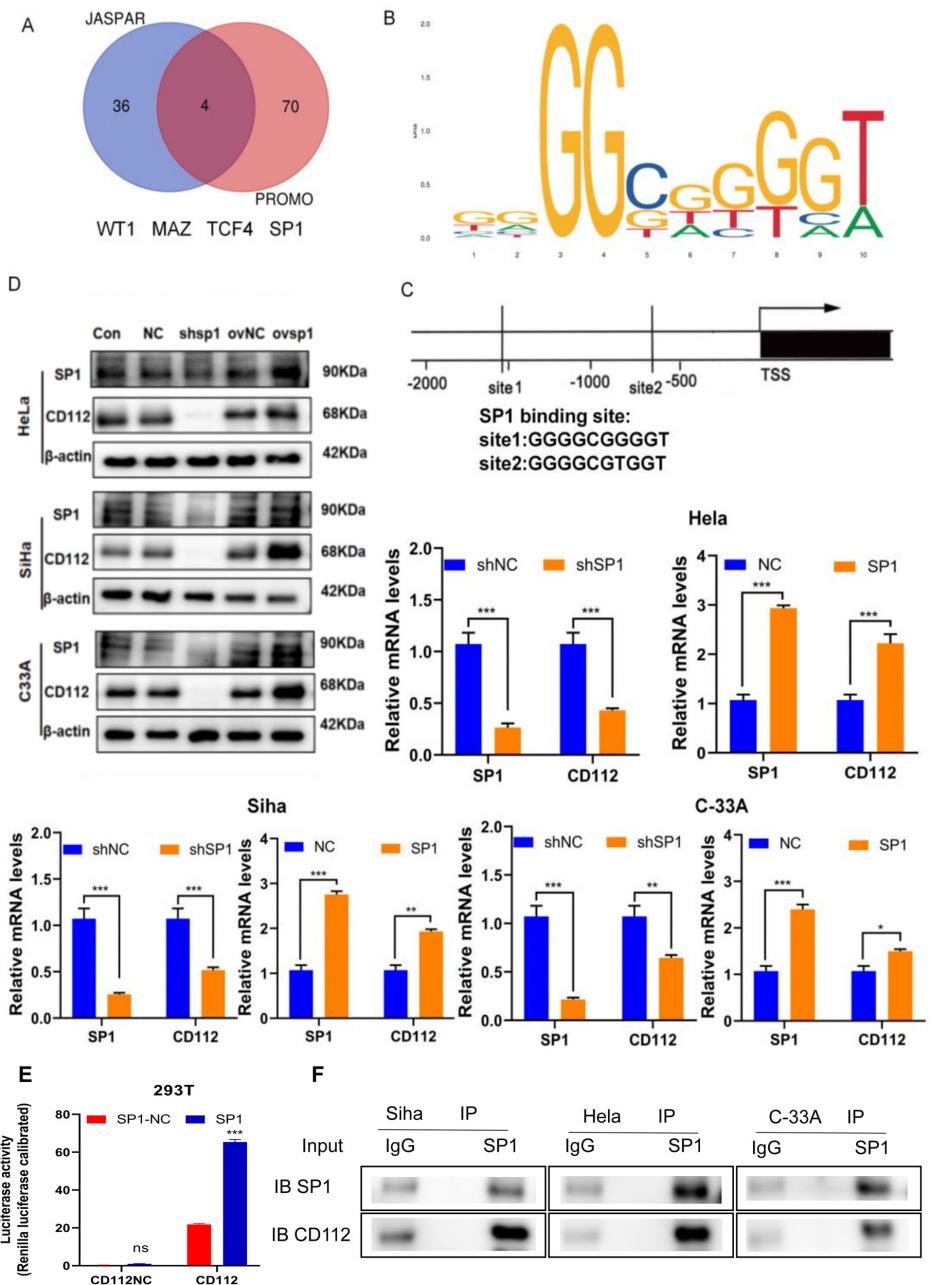
Transcription factor SP 1 promotes CD112 transcription in cervical cancer cells. **A** Venn diagram of the upstream transcription factor of CD112; **B** Sequence map of the transcription factor SP1 binding site; **C** A Schematic representation of the SP1 binding sites on the CD112 promoter; **D** CD112 expression after knockdown and overexpression of SP1 in cervical cancer cells was determined by qPCR and Western blot; **E** Dual-Luciferase Reporter Assay verified the mutual binding of CD112 promoter and SP1 in 293 T cell; **F** Co-IP verified SP1 binding to CD112 in cervical cancer cells. All experiments were performed three times, and data are presented as mean ± SD. ns non significant; *p < 0.05; **p < 0.01; ***p < 0.001.

Then, the promoter sequence of CD112 was searched through the NCBI website, and the transcription factors and binding sites that bound to the promoter of CD112 were predicted by JASPAR and PROMO website. We found four potential candidate transcription factors, namely WT1, MAZ, TCF4 and SP1 (Fig. 7A). Further analysis revealed a binding peak between transcription factor SP1 and CD112 promoter, and identified the sequence with higher scores (Fig. 7B, C). Subsequently, we found that knockdown of SP1 in HeLa, SiHa, and C-33A cells resulted in a significant decrease in CD112 expression by PCR and WB. Conversely, after overexpression of SP1 in the three cell lines, the expression of CD112 was significantly increased (Fig. 7D). This suggests that SP1 can promote CD112 expression at both mRNA and protein levels. Furthermore, we observed that overexpression of viral oncogenic proteins (E6/E7) promoted the upregulation of SP1 and CD112 expression (Figure S5C).

To verify whether SP1 could directly regulate the expression of CD112, we used the dual-luciferase reporter gene assay and found that overexpression of SP1 significantly enhanced the luciferase activity of the wild-type CD112 promoter (Fig. 7E). Secondly, the results of Co-IP experiment showed that transcription factors SP1 and CD112 were bound to each other in HeLa, SiHa and C-33A cells (Fig. 7F).

### Knockdown of SP1 reversed the tumor-promoting effect of CD112 overexpression in CC

To further investigate the effect of SP1 on the biological function of CD112. Reversion assay was used to investigate whether down-regulation of SP1 reversed the CD112-induced proliferation, invasion and metastasis phenotypes of CC cells. The results of the growth curve analysis showed that after knockdown of SP1 in CD112-over-expressing CC cells, the proliferative ability of the CC cells was significantly reduced compared to the control group (Figure S3A). Transwell assays revealed that after SP1 knockdown in CD112-over-expressing CC cells, the invasiveness and metastatic ability of the CC cells were also decreased compared to the control group (Figure S3B). These results suggest that down-regulation of SP1 expression in CC can reverse the malignant biological behaviors, including proliferation, invasion and metastasis induced by CD112 overexpression in CC cells.

### CD112 inhibits the sensitivity of ferroptosis in CC cells by activating the SLC7A11/GPX4 pathway

Furthermore, to explore other potential mechanisms through which CD112 promotes the proliferation, invasion and metastasis of CC cells, we established HeLa-shCD112 cell lines and performed protein quantitative proteomics analysis with the control group. The results revealed distinct protein profiles, with 118 differentially expressed proteins after CD112 knockdown, including 77 upregulated proteins and 41 down-regulated proteins (Figure S4A). Heatmap analysis of the two groups of cells indicated two key ferroptosis-related molecules, PTGS2 and GPX4, which showed significant differences (Figure S4A). This suggests that CD112 may affect ferroptosis in CC cells. To further confirm the impact of CD112 on ferroptosis in CC cells, we performed a joint analysis of the differentially expressed proteins with the ferroptosis online database (FerrDb). The results identified several ferroptosis driver genes (TNFAIP3), marker genes (PTGS2), and suppressor genes (AKR1C2, AKR1C3, AKR1C1, and GPX4) that may be regulated by CD112 (Figure S4C).

The regulation of ferroptosis mainly involves the interplay between the ferroptosis defense system and the ferroptosis execution system. The GPX4-dependent system (SLC7A11-GSH-GPX4 axis) is the most important ferroptosis defense system [22]. We then further validated by PCR that in CD112 knockdown HeLa cells, the expression of PTGS2 was elevated, while the expression of SLC7A11 and GPX4 was decreased, consistent with the proteomics results (Figure S4D). WB revealed that in CC cells with CD112 knockdown, the expression of SLC7A11 and GPX4 was significantly reduced, while the expression of PTGS2 (COX2) and CD71 (TFR1) was significantly increased. In contrast, after CD112 overexpression, the expression of these proteins showed the opposite trend (Figure S4E). Additionally, we assessed the impact of CD112 on intracellular ferrous ions (Fe^2+^) and MDA levels in CC cells. The results showed that after CD112 knockdown, the intracellular Fe^2+^ levels were significantly increased, promoting iron accumulation in the cells, and the MDA levels were significantly elevated, thereby increasing the cells’ sensitivity to ferroptosis. Up-regulation of CD112 reversed these effects. In a cervical cancer cell model with stable overexpression of CD112, we separately knocked down SLC7A11 and then treated the cells with RSL3. The study found that overexpression of CD112 protected cells from RSL3-induced ferroptosis, and this protective effect was reversed when SLC7A11 was simultaneously knocked down. Conversely, in cervical cancer cells with CD112 knockdown and overexpression of SLC7A11, RSL3 treatment similarly revealed that CD112 knockdown enhanced cell sensitivity to RSL3, which could be rescued by overexpression of SLC7A11 (Figure. S4F). These results collectively suggest that CD112 inhibits ferroptosis in CC cells by activating the GPX4-dependent ferroptosis defense system.

RSL3, as a GPX4-targeting inhibitor, can effectively suppress the expression and function of GPX4 [23]. To further investigate the effect of CD112 on ferroptosis in CC cells, we validated the impact of CD112 on the SLC7A11-GPX4 pathway and ferroptosis by co-treating with the ferroptosis inducer (RSL3). After knocking down CD112 in CC cells and treating them with an appropriate concentration of RSL3 for 24 h, we found that the intracellular Fe^2+^ levels were significantly elevated. In contrast, in the three CC cells over-expressing CD112, the Fe^2+^ levels did not show a significant increase. The MDA levels followed the same trend. Therefore, the combination of RSL3 and CD112 knockdown in CC cells produced an additive effect, while RSL3 treatment and CD112 overexpression led to an antagonistic effect. Finally, we performed IHC analysis on mouse tumor tissue samples with CD112 group and control group cells to detect the expression levels of ferroptosis-related proteins (Figure. S4G). The results showed that in tumors from CD112 group, the expression of SLC7A11 and GPX4 proteins was significantly higher than in the control group, while the expression of CD71 and PTGS2 was significantly lower. These results collectively suggest that CD112 inhibits ferroptosis in CC cells by activating the GPX4-dependent ferroptosis defense system, significantly reducing the cells’ sensitivity to ferroptosis.

## Discussion

Patients with metastatic/relapsed cervical cancer have limited treatment options and a very poor prognosis. PD-1 inhibitors have ushered in a new era in the treatment of cervical cancer. However, the response rate of PD-1/PD-L1 inhibitors as monotherapy in cervical cancer is relatively low, resulting in most patients not benefiting from immunotherapy [9, 10]. Current data suggest that monotherapy is unlikely to achieve satisfactory results, while combination therapies with anti-PD-1 or anti-PD-L1 hold more promise for achieving maximum clinical efficacy [24]. Therefore, there is an urgent need to explore new immune checkpoints, accurately identify potential beneficiary populations, and enhance the efficacy of immunotherapy.

In this study, we identified a potential novel immune checkpoint, CD112, in cervical cancer through proteomics combined with a novel list of immune checkpoints. By analyzing 318 cervical cancer specimens, we confirmed tumor core enrichment of CD112 and immune-infiltrated areas CD112R dominance(*P* < 0.05). Additionally, the interaction between CD112 and CD112R, as well as CD112 expression, correlated with the density of PD-1^+^ CD8^+^ T cells in the tumor-infiltrated and tumor-margin regions (*r* = 0.545, *P* < 0.001), and is associated with an unfavorable prognosis. Subsequently, in vitro co-culture models and antibody blockade treatment models in vivo further demonstrated that the combination of CD112 and CD112R creates an immunosuppressive tumor microenvironment, mediating immune evasion. Blocking the CD112/CD112R axis can produce significant anti-tumor effects, indicating that the CD112/CD112R axis may be an effective immunotherapy target in cervical cancer. Finally, we further elucidated the molecular mechanism by which the transcription factor SP1 promotes the transcription of CD112, activating the SLC7A11/GPX4 pathway, which inhibits the sensitivity of cervical cancer cells to ferroptosis

CD112 (PVRL2) is the most highly expressed member of the PVR gene family and is upregulated in various malignant tumors, such as lung cancer, ovarian cancer, and breast cancer [12–14]. Furthermore, an increasing number of studies have confirmed the role of CD112 in tumorigenesis and development, indicating that its overexpression is associated with poor prognosis in patients [25, 26]. In gynecological tumors, CD112 is highly expressed in ovarian cancer and is related to lymph node metastasis and recurrence of ovarian cancer [13]. However, there have been no relevant studies on CD112 in cervical cancer. CD112 is an adhesion molecule primarily located at the adhesion junctions of epithelial cells. Related studies have pointed out that the Nectin family is a key group of cell adhesion molecules that are closely related to cadherins during the formation of adhesion junctions [27, 28]. Cell adhesion junctions are major structures for cell-to-cell adhesion, involving interactions between cell adhesion molecules and transmembrane proteins located on the cell surface. They connect cells in various ways and can participate in signal transduction for cells to detect and respond to changes in their surrounding environment. Alterations in adhesion can disrupt important cellular processes and lead to various diseases, including malignant tumors [29]. Abnormal expression of the Nectin family can result in the loss of cellular stability, enhanced cell proliferation, and migratory capacity, promoting the occurrence and development of malignant tumors [30].

CD112 promotes the proliferation and invasion of cervical cancer cells. In this study, we further explored the underlying mechanisms. We validated through cellular and animal experiments that CD112 activates the SLC7A11-GPX4 pathway, reducing the sensitivity of cells to ferroptosis. Ferroptosis is a novel type of cell death that has been reported in many malignant tumors, such as breast cancer, liver cancer, gastric cancer, colorectal cancer, prostate cancer, glioblastoma, and pancreatic cancer [31–33]. Among these, the GPX4-dependent system (SLC7A11-GSH-GPX4 axis) is the most important defense system against ferroptosis [22]. Combined with the latest research progress, ferroptosis is likely to be a crucial hub in regulating tumor immunotherapy [34]. Furthermore, through molecular interaction experiments, we found that the transcription factor SP1 directly binds to the CD112 promoter and promotes the transcription of CD112. Finally, using rescue experiments, we demonstrated that knocking down SP1 in CD112-over-expressing cervical cancer cells can reverse the phenotypes of CD112 promoting the proliferation, invasion, and migration of cervical cancer cells. SP1 is one of the earliest discovered transcription factors and is a member of the zinc finger family. It is a protein that directly binds to high GC content elements in promoters and regulates the expression of various important genes during cell proliferation and death [35, 36]. Relevant studies have shown that SP1 is highly expressed in various malignant tumors, such as lung cancer, breast cancer, and cervical cancer [37–39]. Additionally, SP1 plays different roles in different tumor cells.

The immune checkpoint CD112 promotes malignant biological behaviors in cervical cancer cells. We have confirmed that it regulates pathways associated with cell proliferation, invasion, and metastasis. However, the role of immune checkpoints and their receptors in the tumor immune microenvironment remains incompletely understood. Our study found that in cervical cancer tissues, the novel immune checkpoint CD112/CD112R interaction inhibits the function of CD8⁺ T lymphocytes. Moreover, in a murine cervical cancer xenograft model, we verified that blocking either CD112 or PD-1 effectively suppresses tumor growth, and the combined blockade of CD112 and PD-1 exerts a significantly stronger anti-tumor effect than CD112 monotherapy. mIHC of mouse tissues further demonstrated that treatment with either a CD112 inhibitor or a PD-1 inhibitor markedly enhanced the quantity and functionality of CD8⁺ T cells in the tumor immune microenvironment. Notably, the proportion of TCF1⁺CD8⁺ T cells, which possess stem-like properties, was also significantly elevated.

The tumor immune microenvironment (TME) profoundly influences tumor initiation, progression, and metastasis. Within the TME, tumor cells and their surrounding milieu frequently secrete a multitude of immunomodulatory molecules that exert either inhibitory or activating effects on immune cell function [40, 41]. CD8⁺ T lymphocytes in the TME are key executors of adaptive immunity, mediating anti-tumor and anti-viral immune responses. However, upon chronic antigen exposure, CD8⁺ T cells can enter a distinct differentiation state: T cell exhaustion, characterized by reduced expression of IL-2, TNF-α, and IFN-γ, and elevated expression of inhibitory receptors such as TIM-3, LAG-3, and PD-1. These exhausted T cells represent a major obstacle to effective anti-viral and anti-tumor immunity during persistent infections and tumor progression [42]. Accordingly, TIM-3⁺CD8⁺ T cells and LAG-3⁺CD8⁺ T cells constitute important components of the exhausted T cell pool. TOX, a key transcription factor, acts as a master regulator driving T cells into and maintaining the exhausted state. High expression of TOX is essential for the commitment to exhaustion, as it establishes and stabilizes the epigenetic and transcriptional program of exhausted T cells by regulating a set of exhaustion-associated genes, including PD-1. Therefore, TOX⁺CD8⁺ T cells represent the core population of deeply exhausted T cells. TCF1, in contrast, is a central marker for stem-like progenitor exhausted T cells. TCF1⁺CD8⁺ T cells exhibit stem-like characteristics and are considered the “reservoir” or “seed cells” for intratumoral effector T cells, capable of continuously replenishing the effector pool depleted by exhaustion, thereby sustaining a durable immune response [43]. A substantial body of research indicates that the presence of an intratumoral TCF1⁺ cell subset is critical for the success of immune checkpoint inhibitors, such as anti-PD-1 therapy. These cells are primary responders to treatment, able to proliferate and differentiate into new effector cells upon therapy, thereby contributing to tumor control [44].

To date, no immunotherapy has been able to completely reverse T cell exhaustion. How to rescue tumor-infiltrating CD8⁺ T lymphocytes from the exhausted state remains a challenging goal. Targeting “second-generation” immune checkpoints is currently a major focus in immunotherapy. Multiple studies have shown that blocking CD112 can significantly enhance anti-tumor immune responses. Recently, Li et al. found that blockade of CD112, whether administered early or late, effectively inhibits tumor growth and prolongs the survival of tumor-bearing mice [45]. Turnis et al. [19] reported that blocking CD112R promotes IFN-γ production by tumor-infiltrating immune cells, with efficacy comparable to blocking TIGIT or PD-1. Murter et al. [20] established melanoma and colon cancer mouse models with CD112R knockout and observed that, compared to wild-type mice, CD112R-deficient mice exhibited suppressed tumor growth and an increased number of tumor-infiltrating immune cells, particularly CD8⁺ T cells, within the tumors.

CD112 emerges as a novel immune checkpoint (IC) in various malignancies, its spatial biology and dual mechanisms in CC progression are unexplored. This study systematically evaluated the spatial distribution of CD112/CD112R axis in cervical cancer tissues, and further analyzing its relationship with prognosis may be meaningful for patient stratification, and patients with high CD112 expression may benefit from combination therapy. Secondly, CD112/CD112R as a potential immunotherapy target is suggested by preclinical animal studies, especially since the combination of PD-1 inhibitors may improve the response rate of immunotherapy. Specifically, the CD112/CD112R axis not only exerts a similar function of PD-1 /PD-L1, exerts an immune escape function by mediating T cell exhaustion, and becomes a dual-effect target by mediating ferroptosis.

In this study, we focused on the expression pattern of CD112/CD112R in cervical cancer, and its mechanism of action in cervical cancer cells, showing similar findings to those in other tumors, and found its dual-target mechanism mediating immunosuppression and abnormal iron metabolism in cervical cancer. And we developed inhibitors targeting CD112 to explore strategies for combination therapy. However, further preclinical and clinical trials are still needed to verify the safety and efficacy.

Several anti-CD112R (PVRIG) monoclonal antibodies are under clinical development for the treatment of advanced solid tumors, including COM701 (NCT03667716) [18]. GSK4381562 (SRF-813, NCT05277051) [46]. and JS009 (NCT05650203) [47]. which are being evaluated either as monotherapy or in combination with PD-1/PD-L1 and/or TIGIT inhibitors to augment anti-tumor immunity. As the first humanized anti-CD112R (PVRIG) antibody developed by Compugen, COM701 has entered phase I/II clinical trials, though the clinical outcomes remain to be disclosed. However, the clinical application of these CD112/CD112R-targeted therapeutics is confronted with potential challenges. CD112 (PVRL2) is widely expressed in normal tissues and plays crucial roles in essential physiological processes such as cell-cell adhesion. Consequently, systemic blockade of the CD112/CD112R axis may induce on-target off-tumor toxicities. To address these challenges and promote the smooth progression of clinical research, strategies focusing on precise tumor targeting should be adopted, including bispecific antibodies or tumor-directed fusion proteins with enhanced selectivity for malignant tissues, as well as Fc-engineered antibodies designed to minimize systemic immune activation. Notably, the combination of COM701 with COM902, an anti-TIGIT monoclonal antibody, has exhibited promising anti-tumor efficacy in in vivo BALB/c mouse models, which supports its potential for clinical translation [48]. Furthermore, PVRL2-targeted antibody-drug conjugates have demonstrated therapeutic anti-tumor activity in ovarian cancer mouse xenograft models [49]. while Fc2-modified anti-PVRL2 antibodies have shown favorable anti-tumor effects with manageable adverse events in non-human primate studies [50].

The main strength of this study is our validated pattern of CD112/CD112R expression in cervical cancer and its underlying molecular mechanism through multiple models. Despite the insights gained from this investigation, several limitations must be acknowledged. Firstly, the reliance on a relatively small sample size for proteomic profiling and spatial mapping may restrict the generalizability of the findings. Secondly, this multicenter study constructed a prognostic prediction model integrating immune microenvironment expression profiles and clinical variables. However, the model lacks an independent external validation set to demonstrate its generalizability. Therefore, future research should collect data from additional centers as external validation sets to further validate the stability of the model results. Thirdly, while the co-culture models and murine experiments provide valuable functional evidence, further validation in diverse clinical contexts is essential to confirm the translational potential of targeting CD112 and its receptor in recurrent/metastatic cervical cancer (R/M CC) treatment. These factors underscore the need for larger, multi-institutional studies to better understand the implications of CD112 in clinical practice.

In conclusion, we found that the novel immune checkpoint CD112/CD112R axis is highly expressed in cervical cancer and associated with a poor prognosis. The transcription factor SP1 promotes CD112 transcription, further activates SLC7A11/GPX4 pathway, and reduces the ferroptosis sensitivity of cervical cancer cells. In addition, CD112 binds to CD112R on the surface of CD8 + T lymphocytes to inhibit the function of CD8 + T lymphocytes and mediate the immunosuppressive TME. Therefore, we found that the CD112/CD112R axis may be a valuable target for immunotherapy and combination therapy of cervical cancer.

## Materials and methods

### Ethics Statement for Fresh Tissue Samples

10 pairs of clinical tissues were obtained from cervical cancer patients who had not undergone preoperative radiotherapy or chemotherapy in PUMCH and immediately stored at −80 °C for proteomics. All of the experimental procedures in this research were approved by the Ethical Committee of Peking Union Medical College Hospital (PUMCH I-23PJ467), and all patients participating in this study have provided written, informed consent for utilization of these clinical materials in research.

### Ethical Statement for cervical cancer tissue microarrays (TMAs)

This study was approved by the ethics committee of Peking Union Medical College Hospital (I-23PJ467) and Zhangzhou Hospital of Fujian Province (2023LWB134). The study complied with the principles outlined in the Declaration of Helsinki. We constructed two cervical cancer TMA of tumor tissues from PUMCH and Zhangzhou Affiliated Hospital of Fujian Medical University between January 2018 and December 2020. A total of 318 paraffin-embedded samples of CC tissues were obtained. The inclusion criteria were as follows: (a) histological identification of CC; 2) complete clinical medical records; 3) typical cancer lesions in pathological sections and FFPE tissues; and 4) complete follow-up data.

### Multiplexed immunofluorescence staining

All the TMAs were deparaffinized in xylene for 30 min and rehydrated in absolute ethyl alcohol for 5 min (twice), 95% ethyl alcohol for 5 min, and 75% ethyl alcohol for 2 min sequentially. Wash the slides with distilled water 3 times. A microwave oven is used for heat-induced epitope retrieval, and during epitope retrieval, the slides were immersed in boiling EDTA buffer (ZLI-9079, ZSBIO, Beijing, China) for 15 min. Antibody Diluent/Block from AlphaX Bio was used for blocking. The mIHC experiments were performed by AlphaPainter X30 and analyzed according to the 5 panels, in which primary antibodies were used as listed in below: CD8 (1:800, ab93278; Abcam), PD-1 (1:800, ab137132; Abcam), CD112 (1:500, ab233384; Abcam), and CD112R (1:200, 21448-1-AP; Proteintech). All the primary antibodies were incubated for 1 h at 37 °C. Then slides were incubated for 10 min at 37 °C. The Alpha TSA Multiplex IHC Kit (AXT37100031, AlphaX Biotech, China) was used for visualization. After each staining cycle, heat-induced epitope retrieval was performed to remove all the antibodies including primary & secondary antibodies. The slides were counterstained for nuclei with DAPI for 5 min and enclosed in Mounting Medium. Multispectral images were scanned with ZEISS AXIOSCAN 7. Cells of interest were quantified using HALO (v.3.5; Indica Labs).

### Cell lines and cell culture

The human cervical cancer cell lines Siha, Caski, HeLa, C33A, ME180, and U14 were purchased from the American Type Culture Collection (Manassas, VA, USA) and cultured according to the respective guidelines. Cervical cancer cell lines were cultured in a humidified incubator with 5% CO_2_ at 37 °C.

### Human-activated CD8^+^ T cell preparation

Peripheral blood mononuclear cells (PBMCs) were isolated from healthy donors using Ficoll-Paque Plus (GE Healthcare, Chicago, IL, USA). CD8 + T cells were isolated from the PBMCs by magnetic bead purification using a Human CD8 + T Cell Enrichment Kit (STEMCELL Technologies, Vancouver, BC, Canada). HLA-A24 phenotype and purity (>90%) were checked by flow cytometry using anti-HLA-A24 (human) monoclonal antibody (mAb)-phycoerythrin (PE) (K0208-5; MBL International) and eF450-labeled antibodies against CD8 (48-0087-42; Thermo Fisher Scientific, Waltham, MA, USA). CD8^+^ T cells were grown in complete RPMI-1640 medium plus interleukin (IL)-2 (10 IU/mL) and activated (1 × 10^5^ cells) by stimulation with plates coated with 2.5 mg/mL of anti-CD3 (OKT3; Thermo Fisher Scientific, Waltham, MA, USA) and 2 mg/mL of anti-CD28 (10F3; Thermo Fisher Scientific, Waltham, MA, USA) in vitro.

### Enzyme-linked immunosorbent assay (ELISA) assay

IL-2, IL-17, IFN-γand TNF-αconcentrations were measured using a IL-2 Quantification Kit (70-EK102HS, Multi science), IL-17 Quantification Kit (70-EK117HS, Multi science), IFN-γQuantification Kit (70-EK180HS, Multi science) and TNF-αQuantification Kit (70-EK182HS, Multi science). ELISA was performed according to the manufacturer’s instructions. All experiments were performed in triplicate.

### Lentivirus infection

Lentiviral vectors for human CD112 overexpression (lenti-CD112) and down-regulation (lenti-CD112), human SLC7A11 overexpression (lenti-SLC7A11) and down-regulation (lenti-SLC7A11) and human SP1 overexpression (lenti-SP1) and down-regulation (lenti-SP1) were obtained from GENE Biotechnology Co. Ltd. (Shanghai). Corresponding empty vectors (lenti-vector and lenti-NC) were used as negative controls. (GENE, Shanghai, China). The transfection was performed with a multiplicity of infection (MOI) of 10-30 in the presence of polybrene (5 µg/mL). Following lentiviral infection, single-cell clones were selected with 3.5 µg/mL puromycin (Sigma-Aldrich) after 2 weeks of incubation. Stably transfected clones were isolated and used for in vitro and in vivo experiments.

### In vivo xenograft model

Female KM mice (5 weeks old) were purchased from Sipeifu (Beijing, China) and housed in an Association for Assessment and Accreditation of Laboratory Animal Care-licensed facility under standardized environmental conditions. The mice received autoclaved food and bedding and acidified drinking water adlibitum. All animal studies were approved by the Institutional Animal Research Ethics Committee of Peking Union Medical College Hospital (No. XHDW-2025-020). All methods were performed in accordance with the relevant guidelines and regulations. For xenograft proliferation studies, U14-NC and U14-CD112 cells (8 × 10^6^ cells) in 200 μL of PBS containing Matrigel were injected subcutaneously into the KM mice (5 weeks old) (*n* = 6 for each group). The tumor growth rate was monitored by measuring tumor diameters every 3 days. The mice were sacrificed at the endpoint, and the xenografted tumors were measured and weighed.

For blocking the CD112/CD112R axis studies, 8 × 10^6^ U14 cells were injected subcutaneously into the left flank of 4 to 6-week-old Kunming female mice (*n* = 24). When tumors reached a size of ≈100 mm^3^, mice were randomly assigned into four different groups with anti-CD112 antibody (10 mg/Kg), anti-PD-1 antibody (Bioxcell, CD279, Clone: RMP1-14) (5 mg/Kg), anti-CD112 antibody (10 mg/Kg) + Anti-PD-1 antibody (5 mg/Kg), and IgG isotype control (Bioxcell) (10 mg/Kg), three times a week for 2 weeks (*n* = 6 for each group). On the third day after treatment, the body weight and growth rate were monitored by measuring tumor diameters thrice weekly. At the endpoint, all of the mice were killed under anesthesia. The tumor tissues of the four groups were collected and fixed in 10% formalin for mIHC.

### Quantitative reverse transcription-polymerase chain reaction (qRT-PCR)

Total RNA was isolated from logarithmically growing cells using the Total RNA Miniprep Kit (Axygen Scientific, Inc., USA) according to the manufacturer’s instructions and reverse-transcribed into cDNA using a qPCR RT Kit (TOYOBO, Shanghai, China) after RNA quantification. qRT-PCR was performed using the THUNDERBIRD SYBR qPCR Mix (TOYOBO, Shanghai, China) on an ABI PRISM 7500HT instrument (Applied Biosystems). The expression level of the target mRNA was normalized to the glyceraldehyde-3-phosphate dehydrogenase (*GAPDH*) expression level and was determined according to the 2^*−ΔΔCT*^ method.

### Western blot

Protein samples extracted from logarithmically growing cells were separated by gel electrophoresis, and transferred onto nitrocellulose membranes (Invitrogen, Carlsbad, USA). Subsequently, the membrane was blocked for 1 h and incubated overnight at 4 °C with primary antibodies: CD112 (1:2,000, ab314230), SP1 (1:1,000, ab124804), CD71 (1:1,000, ab214039), PTGS2 (COX-2) (1:2,000, ab179800), SLC7A11 (1:1,000, ab307601), and GPX4 (1:2,000, ab125066). Finally, the membrane was incubated with secondary antibody Horseradish peroxidase (HRP)-linked anti-mouse (1:2000, ab6728) and anti-rabbit (1:2000, ab6721; Abcam, Cambridge, UK) immunoglobulin G (IgG), which were used as secondary antibodies at room temperature for 1 h, and the Odyssey® Imaging System (LI-COR, USA) was used to visualize and analyze the proteins.

### Cell counting Kit-8 (CCK-8) assays

1000–1500 stably transfected cells were seeded onto a 96-well plate and cultured for 4 h for attachment. Subsequently, the culture solution was replaced with a solution containing CCK-8 reagent, and the optical density (OD) value was determined.

### 5-ethynyl-2′-deoxyuridine assay

Again, as described above, first resuspend CC cells in culture medium and inoculate in 96-well plates at 10,000 cells per 100 µL. The cells were given 8 h to adhere to the surface, followed by a 48-h treatment with the appropriate concentration. Cells were incubated with 5-ethynyl-2′-deoxyuridine (EdU, 10 μM, UE, China) for 2 h, followed by fixation using 4% paraformaldehyde for 20 min. They were then permeabilized with 0.3% Triton X-100 for 30 min and thoroughly washed with BSA. Then, EdU staining was performed by incubating cells for 1 h, followed by DAPI staining for nuclear visualization. Ultimately, images were captured with a fluorescence microscope, and the count of Edu-positive cells was assessed through ImageJ software.

### Wound-healing assay

Stably transfected cells were seeded in a 6-well plate at 30 × 10^4^ cells/well and cultured until cell fusion occurred. A 10-µL pipette tip was used to scratch a straight cut at the bottom of the plate. The floating cells were washed away with phosphate-buffered saline (PBS) and cultured in serum-free medium, and wound closure was photographed at 0 and 24 h.

### Transwell invasion assay

Matrigel-coated (BD Biosciences, Franklin Lakes, NJ, USA) Transwell plates were used to examine the invasion abilities of the cells. Cells were inoculated into the upper chamber of the transwell, and serum-free medium was added. Normal media was injected into the plate wells. After a 48 h incubation period, cells from the upper layer filter were discarded, and the cells in the bottom layer were fixed, stained, and counted.

### Dual-luciferase reporter assay

The purpose of the dual-luciferase reporter gene assay was to analyze the transcriptional regulation of transcription factors on-target genes. The full-length promoter of CD112 carrying mutant or wild-type sequences was cloned into pGLO4.10 vectors (Promega, Madison, WI,

USA) and co-transfected with a SP1 overexpression vector or mock vector into 293 T cells, using Lipofectamine TM 2000 (Invitrogen, CA, USA). After 48 h of culture, firefly and Renilla luciferase activities were measured using a dual-luciferase reporter gene assay system (Beyotime, Shanghai, China) in accordance with the manufacturer’s protocols.

### Co-immunoprecipitation (IP) assay

Cells were harvested and then lysed in 500 µL co-IP buffer containing a protease inhibitor cocktail (Sigma-Aldrich). After centrifugation, cell lysates were collected and pre-cleared by incubating with 20 µL immobilized protein A/G beads for 1 h at 4 °C. The beads were then discarded using a magnetic frame, and the lysates were incubated with primary antibody or control immunoglobulin (Ig)G on a rotator at 4 °C overnight. On the following day, 20 µL of immobilized protein A/G beads were added to precipitate the protein complex at 4 °C for 4 h. Subsequently, samples were washed five times, the beads were boiled in loading buffer, and the proteins were prepared for Western blot as described above.

### Proximity ligation assay

The DuoLink® In Situ Red Starter Kit Mouse/Rabbit (DUO92101, Sigma-Aldrich, Darmstadt, Germany) was used to detect interacting proteins. The paraffin-embedded cervical cancer tissue microarrays were deparaffinized. Then, antigen retrieval was performed using sodium citrate. Then slides were blocked with Duolink Blocking Solution in a pre-heated humidified chamber for 30 min at 37 °C. The primary antibody to detect CD112 and CD112R was added to the slides and incubated overnight at 4 °C. Then slides were washed with 1×Wash Buffer A and subsequently incubated with the two PLA probes (1:5 diluted in antibody diluents) for 1 h, then the Ligation-Ligase solution for 30 min, and the Amplification-Polymerase solution for 100 min in a pre-heated humidified chamber at 37 °C. Before imaging, slides were washed with 1×Wash Buffer B and mounted with a cover slip using Duolink In Situ Mounting Medium with DAPI. Fluorescence images were acquired using a Leica TCS SP8 confocal microscope.

### Statistical analysis

Statistical analyses were performed using SPSS software (version 22.0; IBM Corporation, Armonk, NY, USA) and GraphPad Prism version 8.0 (GraphPad Software, La Jolla, CA, USA). Continuous variables are expressed as mean (standard deviation), and categorical variables are expressed as numbers. Differences between groups were assessed using the *t*-test or chiq2 test, as appropriate. Overall survival (OS) was defined as the time from surgery to death from any cause. Disease-free survival (DFS) is the time from surgery to recurrence or death from any cause. Survival curves were estimated using the Kaplan-Meier method, and the log-rank test was used to determine statistical significance. In the multivariate Cox regression analysis, the model was adjusted for key clinicopathological variables, including age, FIGO stage, lymph node metastasis status, lymphovascular invasion, tumor size, and depth of myometrial invasion. Images from all representative histological experiments, western blot, and IF were obtained at least three times independently. All tests were 2-sided with a significance level of *P* < 0.05. *P < 0.05; **P < 0.01; ***P < 0.001.

## Supplementary information

Supplementary Figures and Tables

Supplementary File 2 Original Western blot images

## Data Availability

The data that support the findings of this study are available from the corresponding author upon reasonable request.
