# Supplementary material for: Spatiotemporal mapping of CD112 unveils its dichotomous role in T-cell exhaustion and ferroptosis crosstalk in cervical cancer
Source: Cell Death Dis. 2026 May 27;17(1):655. doi: 10.1038/s41419-026-08902-y (PMC13396493; doi:10.1038/s41419-026-08902-y)

Full blots for Fig 7D

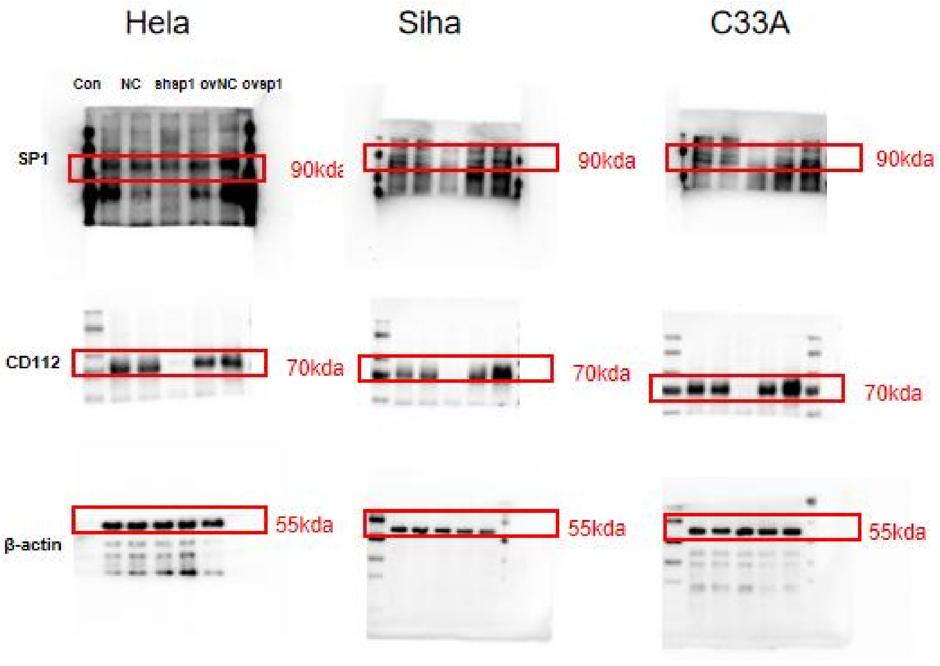

Full blots for Fig 7F

CD112

70kda

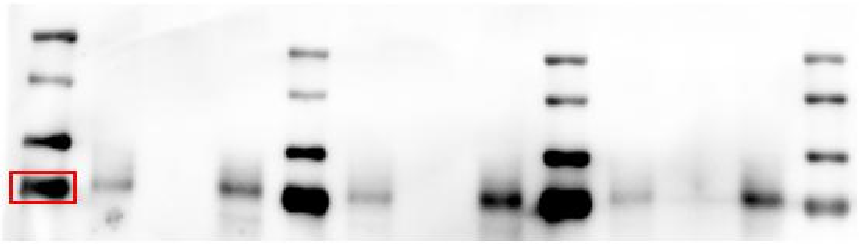

SP1

90kda

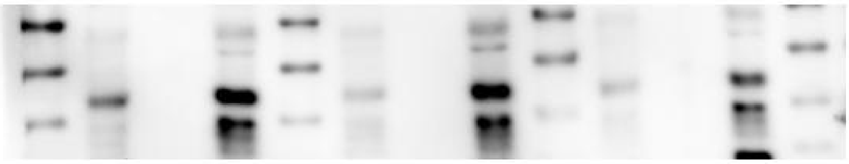

Full blots for Fig S2A

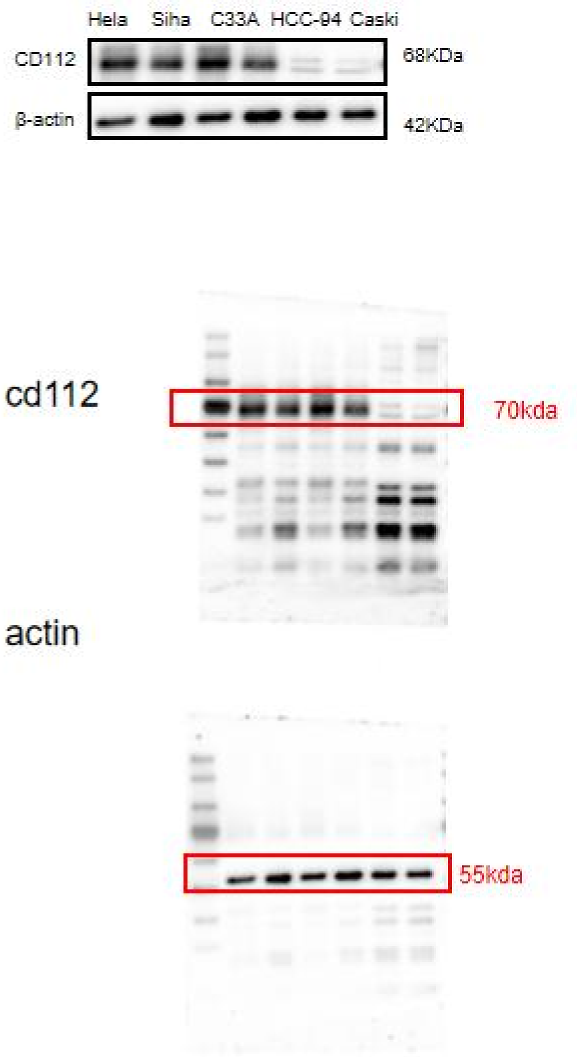

Full blots for Fig S2B

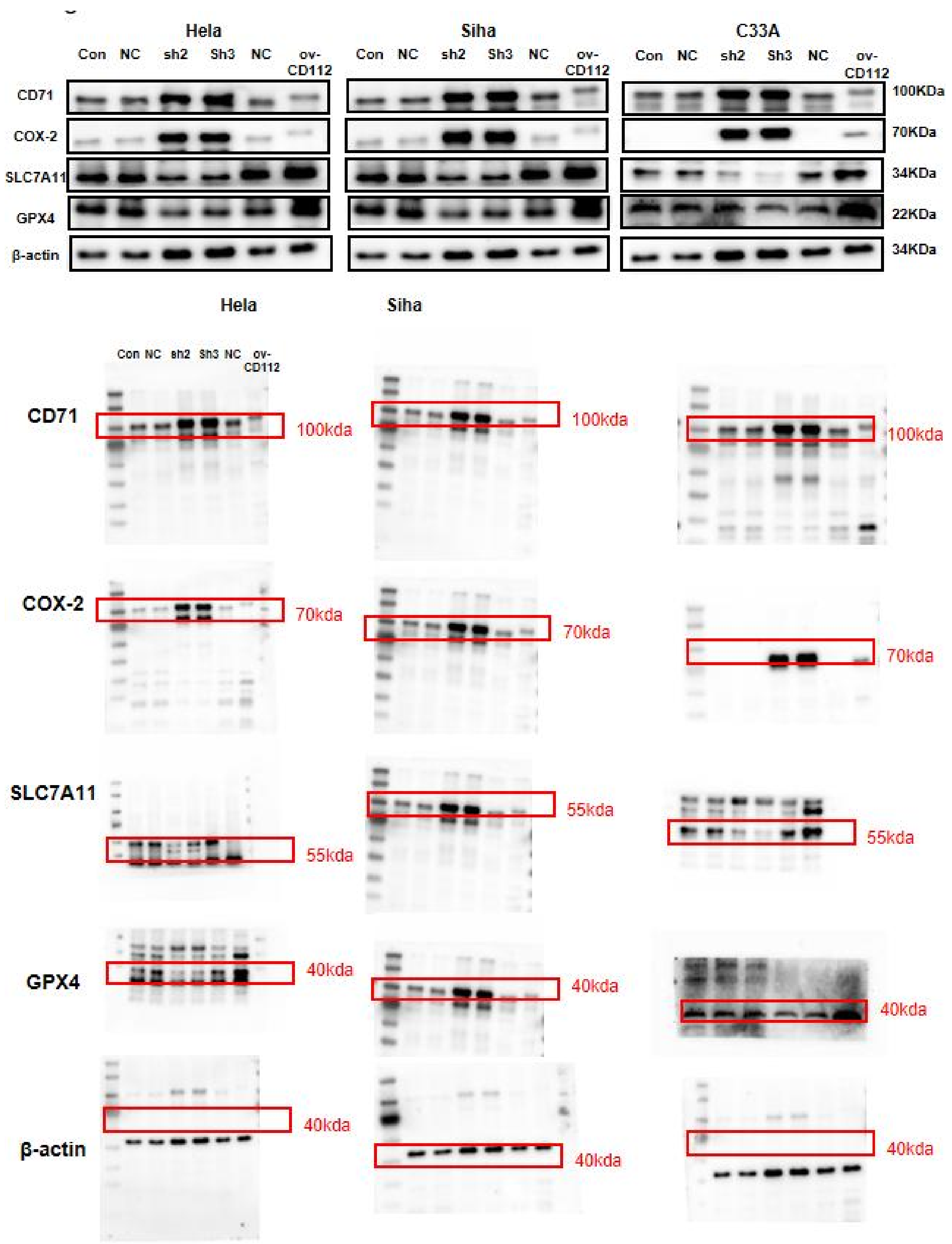

Full blots for Fig S4F

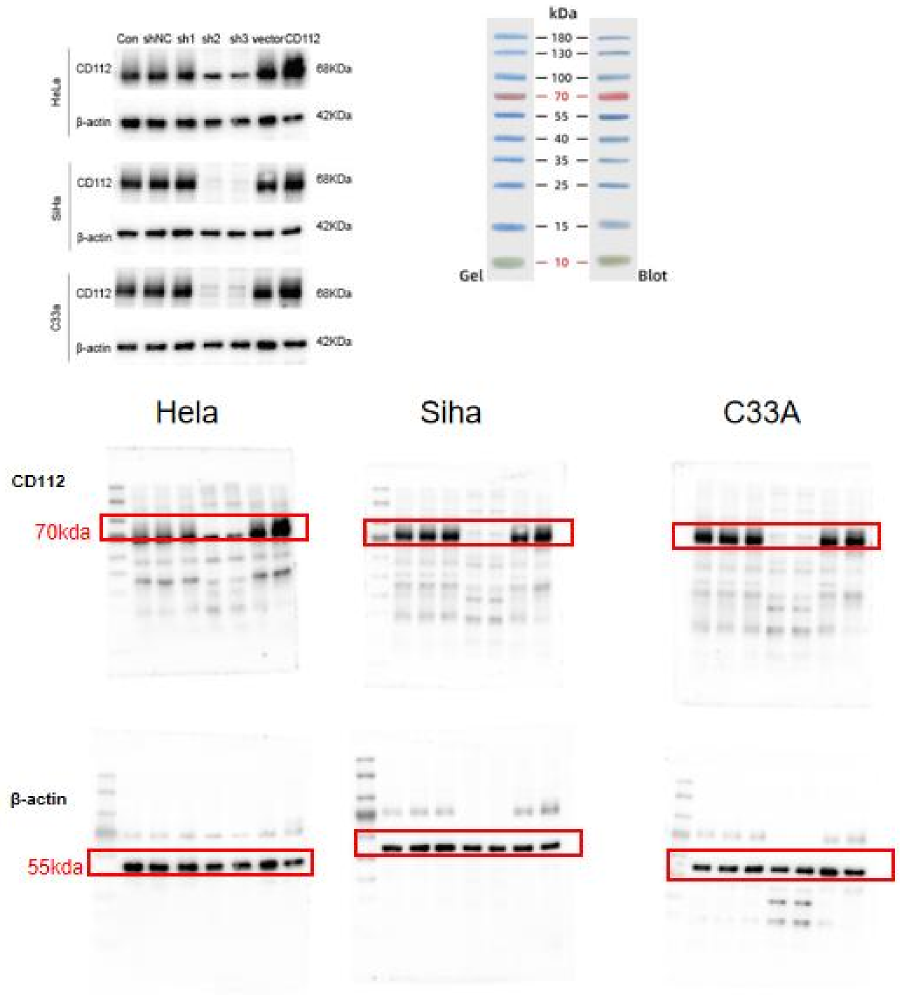

Full blots for Supplementary Fig S5A

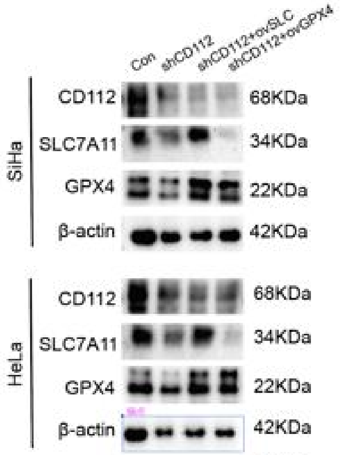

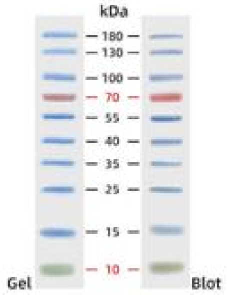

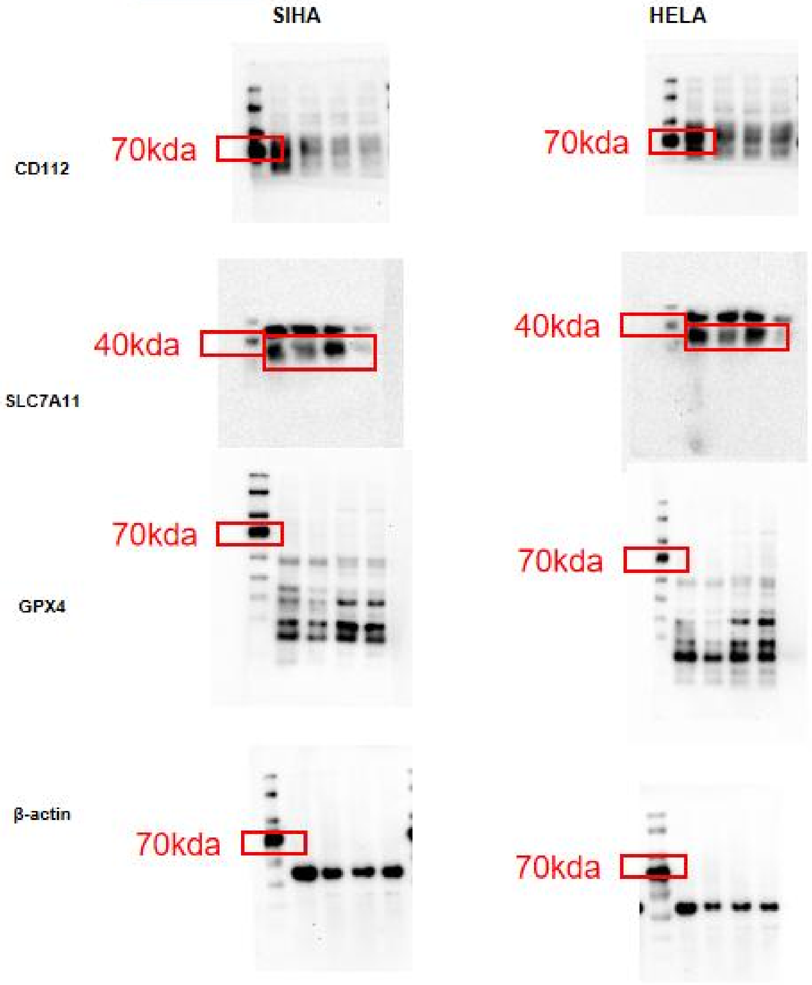

Full blots for Supplementary Fig S5A

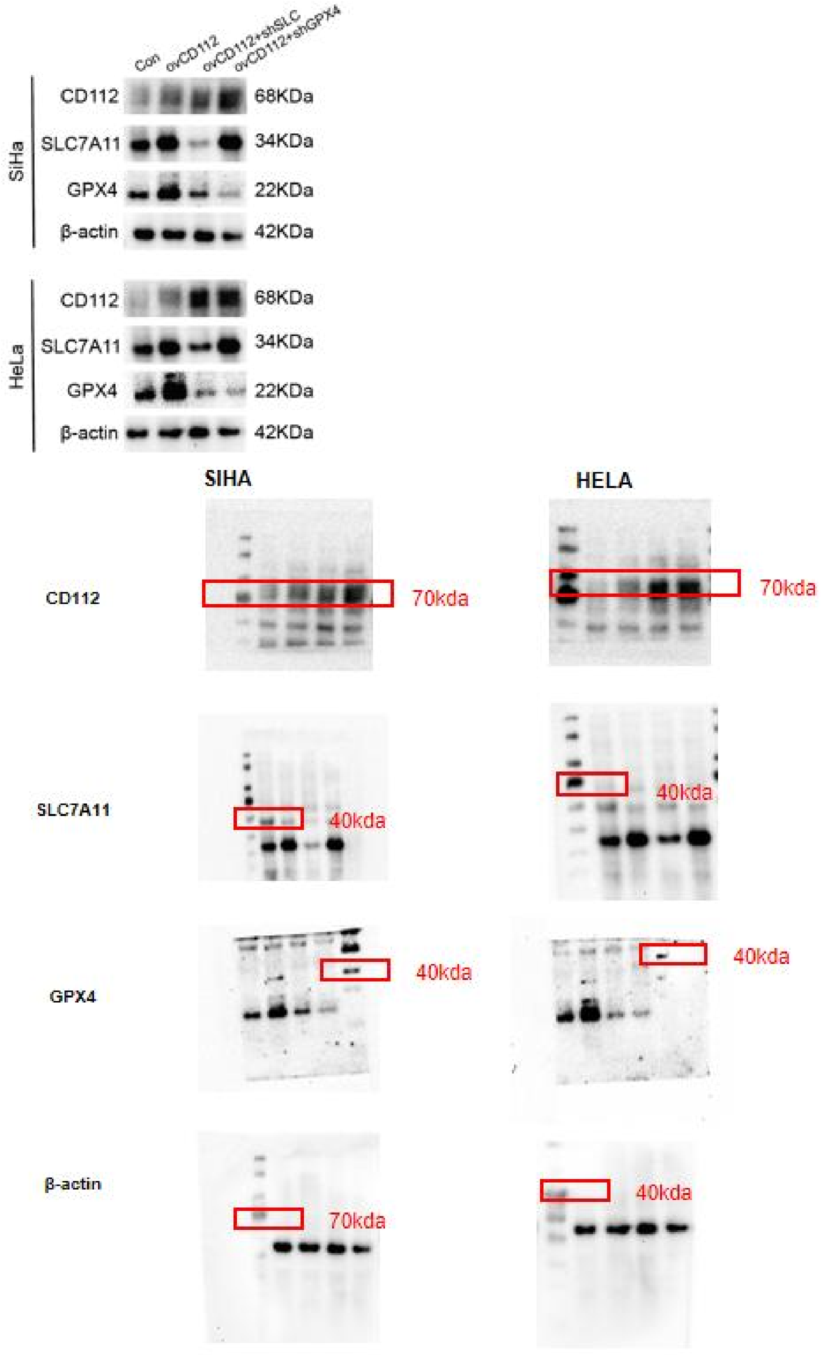

Full blots for Supplementary Fig S5C

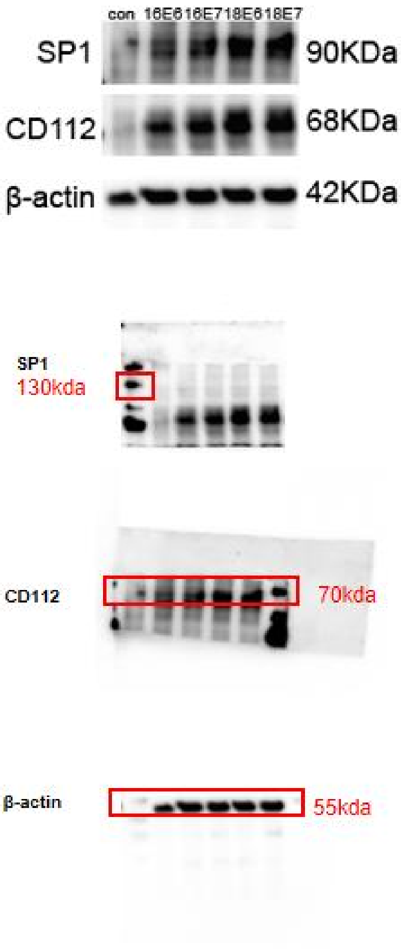

Supplement: Supplementary file 2 — Original Western blot images [file 41419_2026_8902_MOESM2_ESM.pdf]
