# Supplementary material for: Spatiotemporal mapping of CD112 unveils its dichotomous role in T-cell exhaustion and ferroptosis crosstalk in cervical cancer
Source: Cell Death Dis. 2026 May 27;17(1):655. doi: 10.1038/s41419-026-08902-y (PMC13396493; doi:10.1038/s41419-026-08902-y)
Supplement: Supplementary file 1 — Supplementary Figures and Tables [file 41419_2026_8902_MOESM1_ESM.docx]

Supplementary Figures and Tables

**Spatiotemporal Mapping of CD112 Unveils Its Dichotomous Role in T-cell Exhaustion and Ferroptosis Crosstalk in Cervical Cancer**

Lihua Chen^1,2^†, Wenyu Lin^3,4^†, Yuxuan Huang^3,4^†, Chen Li^1,2^, Ming Du^1,2^, Yang Liu^1,2^，Weidi Wang^1,2^, Duancheng Tian^1,2^, Pengming Sun^3,4*^, Yang Xiang^1,2*^

^*^**Address correspondence to:** Yang Xiangy (xiangy@pumch.cn).

**Supplementary Figure**

**Figure S1. Protein profile of the cervical cancer**

A. PCA plots of cervical cancer samples from different groups； B. Volcano plot of differential proteins in cervical cancer and paired adjacent tissues; C. Bar chart of differential protein of cervical cancer and paired adjacent tissues; D. GO classification radar map of differential proteins; E. KEGG pathway enrichment analysis of the differential proteins.

**Figure S2. Construction of Cell Models**

A. WB verified the basic expression level of CD112 in cervical cancer cells; B. WB and qRT‒PCR assays showing the mRNA and protein levels of CD112 after CD112 knock down or over-expression in three cervical cancer cells; C. IC50 experiments explored the concentration and duration time of RSL 3 drugs in cervical cancer cells.

All experiments were performed three times and data are presented as mean±SD. ns non significant; *p<0.05; **p<0.01; ***p<0.001.

**Figure S3. SP 1 promotes CD112 transcription and enhances cervical cancer cell proliferation, invasion and metastasis**

A.Growth curve assay based on CCK8 analysis; interaction effects between shSP1 and CD112 on CC cell proliferation. B. Representative images and statistical analysis of Transwell assay; interaction effects between shSP1 and CD112 invasion and migration (scale bars = 100 μm)；

All experiments were performed three times and data are presented as mean±SD. ns non significant; *p<0.05; **p<0.01; ***p<0.001.

**Figure S4. CD112 inhibits the sensitivity of ferroptosis in cervical cancer cells by activating the SLC7A11/GPX4 pathway**

A.4D Label-free proteomics was used to select the differential proteins regulated by CD112; B. The Bar charts and volcano charts of the differential proteins; C. Venn diagram for the joint analysis of the differential protein and ferroptosis online database (FerrDb); D. qPCR was used to find the stream mediated ferroptosis pathway after CD112 knockdown in cervical cancer cells; E. Western blot was used to demonstrate the expression of PTGS2, CD71, SLC7A11 and GPX4 in cervical cancer cells; F. Fe^2+^ concentration and cell membrane lipid peroxidation level (MDA) were detected in cervical cancer cells after knockdown CD112, over-expression CD112 and combination of ferroptosis inducer (RSL3), respectively; G. IHC staining of CD112, PTGS2，CD71，SLC7A11 and GPX4 expression in mouse tissue(scale bars=100 μm);

All experiments were performed three times and data are presented as mean±SD. ns non significant; *p<0.05; **p<0.01; ***p<0.001.

**Figure S5. Construction of rescue cell models and validation of the relation with CD112 expression and HPV status**

A. WB assays showing the protein levels of CD112, SLC7A11 and GPX4 after in cervical cancer cells; B. To analyze the relationship between different HPV genotypes and CD112 expression in a cohort of cervical cancer patients; C. WB assays showing the protein levels of CD112 and SP1 after over-expression E6/E7 in Hacat.

The data are presented as mean±SD. ns non significant; *p<0.05; **p<0.01; ***p<0.001.

Table S1 The list of novel immune checkpoints

| ICs | Immunological effect | Distribution |
| --- | --- | --- |
| CTLA-4 | Co-suppression | T cells and NK cells |
| PD-1 | Co-suppression | T cells and NK cells |
| PD-L1/2 | Co-suppression | Tumor cells |
| LAG-3 | Co-suppression | Antigen presenting cells |
| TIM-3 | Co-suppression | T cells and NK cells |
| TIGIT | Co-suppression | T cells and NK cells |
| CD155 | Co-suppression | Tumor cells |
| CD112 | Co-suppression | Tumor cells |
| CD96 | Co-suppression | T cells and NK cells |
| CD112R | Co-suppression | T cells and NK cells |
| B7-H3 | Co-suppression | Tumor cells |
| KIR | Co-suppression | NK cells |
| VISTA | Co-suppression | T cells and NK cells |
| BTLA | Co-suppression | T cells and NK cells |
| Sigec-15 | Co-suppression | Macrophage |
| ICOS | Co-stimulatory | T cells and NK cells |
| CD40 | Co-stimulatory | Antigen presenting cells |
| 4-1BB | Co-stimulatory | T cells and NK cells |
| CD28 | Co-stimulatory | T cells |
| OX40 | Co-stimulatory | T cells and NK cells |
| IDO | Co-suppression | Macrophage and DC cells |
| GITR | Co-suppression | T cells and NK cells |
| CD27 | Co-stimulatory | T cells and NK cells |
| CD47 | Co-suppression | Macrophage |
| CD70 | Co-stimulatory | Antigen presenting cells |

Table S2 Characteristics of the patients of TMA cohort

| Characteristics | Number of cases (%) |
| --- | --- |
| Age (years) | 57.1±9.9 |
| FIGO stage（2018） | |
| I | 265（83.3%） |
| II | 53（16.7%） |
| Pathology type | |
| SCC | 254（79.9%） |
| AC | 53（16.7%） |
| ASC | 11（3.5%） |
| Tumor size | |
| ≤2cm | 143（45.0%） |
| ≤4cn | 155（48.7%） |
| ＞4cm | 20（6.3%） |
| Muscle infiltration＞50% | 132（41.5%） |
| LVSI | 112（35.2%） |

SCC, squamous carcinoma of the cervix; AC, adenocarcinoma; ASC, adenosquamous carcinoma;
